# Safe delivery of a highly toxic anthracycline derivative through liposomal nanoformulation achieves complete cancer regression

**DOI:** 10.1186/s12943-025-02444-1

**Published:** 2025-10-27

**Authors:** András Füredi, Szilárd Tóth, Kristóf Hegedüs, Pál T. Szabó, Anikó Gaál, Gergő Barta, Lívia N. Naszályi, Krisztina Kiss, Kata Bölcskei, Zoltán Szeltner, Eszter Bajtai, Balázs Gombos, Dániel Kiss, Mihály T. Cserepes, Attila Kiss, Peter Pokreisz, Lukas Kenner, Sandra Högler, Csaba Magyar, Jamie D. Cowles, Agnes Csiszar, József Tóvári, Dávid Szüts, Zsuzsanna Helyes, Zoltán Varga, Gábor Mező, Gergely Szakács

**Affiliations:** 1 Institute of Molecular Life Sciences, Center of Excellence of the Hungarian Academy of Sciences, HUN-REN Research Centre for Natural Sciences, Budapest, H-1117 Hungary; 2 Institute of Technical Physics and Materials Science, HUN-REN Centre of Energy Research, Budapest, H-1121 Hungary; 3 National Laboratory for Drug Research and Development, Budapest, H-1117 Hungary; 4 Physiological Controls Research Center, University Research and Innovation Center, Óbuda University, Budapest, H-1034 Hungary; 5 Organocatalysis Research Group, Institute of Organic Chemistry, HUN-REN Research Centre for Natural Sciences, Budapest, H-1117 Hungary; 6 Department of Organic Chemistry, Institute of Chemistry, Faculty of Science, ELTE Eötvös Loránd University, Budapest, H-1117 Hungary; 7 Centre for Structural Science, HUN-REN Research Centre for Natural Sciences, Budapest, H- 1117 Hungary; 8 Institute of Materials and Environmental Chemistry, HUN-REN Research Centre for Natural Sciences, Budapest, H-1117 Hungary; 9 HUN-REN-ELTE Research Group of Peptide Chemistry, Budapest, H-1117 Hungary; 10 Department of Pharmacology and Pharmacotherapy, Medical School, University of Pécs, Pécs, H-7624 Hungary; 11 Semmelweis University Doctoral School, Budapest, H-1085 Hungary; 12 John von Neumann Faculty of Informatics, Óbuda University, Budapest, Hungary; 13 Department of Experimental Pharmacology and the National Tumor Biology Laboratory, National Institute of Oncology, Budapest, Hungary; 14 KINETO Lab Ltd, Budapest, H-1037 Hungary; 15 Center for Biomedical Research and Translational Surgery, Medical University of Vienna, Vienna, Austria; 16 Clinical Institute of Pathology, Department for Experimental and Laboratory Animal Pathology, Medical University of Vienna, Vienna, Austria; 17 Unit of Laboratory Animal Pathology, University of Veterinary Medicine Vienna, Vienna, Austria; 18 Center for Cancer Research, Medical University of Vienna, Vienna, Austria; 19 HUN-REN-PTE Chronic Pain Research Group, University of Pécs, Pécs, H-7624 Hungary; 20 PharmInVivo Ltd, Pécs, H-7629 Hungary; 21 Department of Physical Chemistry and Materials Science, Budapest University of Technology and Economics, Budapest, H-1111 Hungary

**Keywords:** Nanomedicine, Chemotherapy, Drug resistance, Cancer eradication, Mouse models of cancer, Anthracycline

## Abstract

**Background:**

Chemotherapy remains the cornerstone of cancer treatment despite its well-documented challenges, including toxic side effects and drug resistance. Here, we demonstrate that a novel, highly toxic, daunosamine-modified derivative of daunorubicin (2-pyrrolino-daunorubicin, PyDau) can be safely administered to mice when encapsulated in liposome.

**Methods:**

PyDau was synthesized from daunorubicin in a one-step reaction. Its increased in vitro cytotoxicity was confirmed across 42 human cell lines representing 12 cancer types, including multidrug resistant cells. The activity profile of this new derivative was analyzed in the context of 13 commonly used cancer drugs across a panel of lymphoblast cell lines missing individual components of DNA-repair enzymes. To enable in vivo application, PyDau was encapsulated in pegylated liposome, resulting in liposomal PyDau (LiPyDau). In vivo efficacy of LiPyDau was evaluated in three allograft models (melanoma, breast, lung), a xenograft model (uterine sarcoma), a patient-derived xenograft model (lung), and a genetically engineered mouse model of mammary cancer, including two models of drug resistance.

**Results:**

While PyDau exhibited up to 1000-fold greater cytotoxicity than daunomycin and doxorubicin against cancer cell lines, its in vivo application was hindered by an extremely narrow therapeutic window. Liposomal nanoformulation mitigated the limiting toxicity, allowing LiPyDau to be tested in preclinical allograft and xenograft mouse models. LiPyDau demonstrated robust efficacy across all models including multidrug-resistant cancer, completely eradicating tumors in a genetically engineered mouse model of triple-negative breast cancer. LiPyDau exerts its anticancer effect through a unique mechanism involving the crosslinking of complementary DNA strands, resulting in irreversible DNA damage.

**Conclusion:**

Liposomal formulations of extremely cytotoxic anthracycline analogs, such as LiPyDau, represent a promising and highly effective therapeutic approach for combating drug resistant cancer.

**Supplementary Information:**

The online version contains supplementary material available at 10.1186/s12943-025-02444-1.

## Background

Despite the advent of targeted therapies, many traditional yet effective drugs remain integral to clinical practice [1]. Rather than being replaced by modern therapies, chemotherapy continues to be widely used, especially in combination regimens. In particular, anthracycline analogues are among the most used chemotherapeutics, listed by WHO as essential medicines for cancer treatment. Daunorubicin (DAU) is primarily used for the treatment of acute leukemias; doxorubicin (DOX) is indicated in lymphomas, sarcomas, and a broad spectrum of solid tumors, including breast, lung, bladder, bone, and cervical cancers [2]. Despite their therapeutic value, the clinical efficacy of anthracyclines is significantly limited by resistance and severe cardiotoxicity, necessitating the development of novel analogues [3]. Resistance to anthracyclines can be attributed to variations in absorption, metabolism, and delivery of drugs to target tissues. Additionally, cellular mechanisms of resistance stem from preexisting genetic heterogeneity [4], adaptive changes [5] or mutations induced by toxic chemotherapy, resulting in the activation of DNA damage pathways, evasion of apoptosis, or increased drug efflux by ABCB1/P-glycoprotein [6].

High mortality rates due to side effects and resistance to treatment underscore the urgent need to improve treatment efficacy and patient outcomes. It has recently been proposed that patients diagnosed with aggressive cancers could benefit from more intense cytotoxic treatments to reduce tumor heterogeneity and minimize the likelihood of drug resistance [4]. However, increasing the dose or frequency of treatment cycles with currently used chemotherapeutic compounds often poses significant safety risks for patients [7]. As an alternative, various strategies have been proposed to increase the potency of anthracyclines, including the enhancing of uptake or inhibition of efflux by cancer cells [8], encapsulation [9], or conjugation with antibodies, peptides, or synthetic polymers [10, 11]. Chemical modifications were also reported to significantly increase the in vitro cytotoxicity of DOX against cancer cells [2, 12]. However, only a few analogues have achieved clinical approval, as addressing all the major limitations of anthracyclines—such as simultaneously enhancing anticancer activity, reducing cardiotoxicity, and mitigating resistance—has proven to be a significant challenge.

Conversion of DOX to 3’-deamino-3’-(2’’-pyrroline-1’’-yl)doxorubicin (PyDox) yielded a highly potent derivative [13]. While PyDox bypasses multidrug resistance [14], due to its extremely narrow therapeutic window, this superactive analog was not suitable for clinical development [15, 16]. Here we show that liposomal formulation of 2-pyrrolino-daunomycin (PyDau), a highly toxic derivative of DAU, can be safely administered to mice to achieve dramatic anticancer effects.

## Methods

### Materials

Ultrapure water (Milli-Q, Millipore, Molsheim, France) was used for the synthesis throughout the experiments. Synthetic high-purity hydrogenated soybean phosphatidylcholine (HSPC), 3β-Hydroxy-5-cholestene (cholesterol), 1,2-distearoyl-sn-glycero-3-phosphoethanolamine-N-[amino(polyethylene glycol)-2000] (ammonium salt) (DSPE-PEG 2000) and 1,2-distearoyl-sn-glycero-3-phosphoethanolamine-*N*-[biotinyl(polyethylene glycol)-2000] (ammonium salt) (DSPE-PEG-2000) were obtained from Avanti Polar Lipids (Alabaster, AL, USA) or Merck Life Science Kft. (Merck KGaA, Darmstadt, Germany). Chloroform (LiChrosolv^®^, Sigma-Aldrich), hydrochloric acid (1 M, Reag. Ph Eur, Reag. USP, Titripur^®^, Supelco), sodium hydroxide (1 M, Reag. Ph Eur, Reag. USP, Titripur^®^, Supelco), L-histidine ((S)-2-Amino-3-(4-imidazolyl)propionic acid, BioUltra, ≥ 99.5%, Sigma-Aldrich), D-(+) saccharose (puriss, PH. EUR, Sigma-Aldrich), ammonium sulfate (Molecular Biology Tested, ≥ 99%, Sigma-Aldrich) was purchased from Merck Life Science Kft (Budapest, Hungary). All buffers used were filtered through 0.22 μm filters (Steritop Vacuum Driven Disposable Filtration System, Merck KGaA or Acrodisc Syringe Filter, PALL Life Sciences, Pall Corporation, New York, USA). Mini-PROTEAN TBE-Urea polyacrylamide (PA) gels (10%) and acrylamide solution (29:1) was purchased from BioRad, GelRed from Biotium. Alkaline (3%) agarose gels were prepared in the laboratory.

### Preparation of LiPyDau

PyDau containing liposomal formulation (LiPyDau) was prepared by the lipid film hydration and extrusion method. The lipid mixture containing HSPC, cholesterol, DSPE-PEG2000 was dissolved in chloroform and dried to a thin lipid film under a stream of N_2_ gas, followed by incubation overnight under vacuum to remove residual solvent. Next, 0.25 M, pH = 6.5 ammonium sulfate solution was used to hydrate the lipid films to gain a total lipid concentration of 16 mg/mL (9.6 mg/mL HSPC, 3.2 mg/mL cholesterol, and 3.2 mg/mL DSPE-PEG-2000, and mixed with a magnetic stirrer at 60 °C for 30 min. The resulting multilamellar vesicle (MLV) suspension was subjected to five cycles of freeze-and-thaw (5 min. each, freezing in liquid nitrogen, and thawing at 60 °C) before being extruded 13 times at 60 °C through a 100 nm polycarbonate membrane filter (Whatman, Springfield Mill, UK) using a LIPEX^®^ Extruder (LIPEX^®^ Biomembranes, Burnaby, BC, Canada). The suspending medium of the liposomes was changed to L-histidine/saccharose buffer (10 mM/10%, pH = 6.5) using a PD-10 column (contain Sephadex G-25 medium, Cytiva, Amersham Place Little Chalfont, England) following the manufacturer’s instructions. Next PyDau-hydrochloride (2 mg/mL stock solution in 0.9% NaCl infusion (B. Braun, Budapest, Hungary) was added to the liposomes (1.5 mL liposome sample + 1.1 mL 2 mg/mL PyDau solution), followed by stirring for 1 h at 60 °C. The unencapsulated PyDau was removed using PD-10 desalting columns. The purification step was repeated two times to ensure that free PyDau was removed completely.

### Characterization of LiPyDau

The morphology of liposomes was observed with freeze-fracture combined transmission electron microscopy (FF-TEM). LiPyDau was mixed with glycerol (Sigma-Aldrich, Hungary) which is used as a cryoprotectant at a 3:1 sample-to-glycerol volume ratio. Approx. 2 µL of the different liposomes was pipetted onto a gold sample holder and frozen by placing it immediately into partially solidified Freon for 20 s. Fracturing was performed at − 100 °C in a Balzers freeze-fracture device (Balzers BAF 400D, Balzers AG, Liechtenstein). The replicas of the fractured surfaces were made by platinum-carbon evaporation and then cleaned with a water solution of surfactant and washed with distilled water. The platinum-carbon replicas were placed on 200 mesh copper grids and examined in a MORGAGNI 268D (FEI, The Netherlands) transmission electron microscope.

Microfluidic resistive pulse sensing (MRPS) was used to determine the liposome concentration and size distribution of LiPyDau. MRPS is the nanoscale implementation of the Coulter principle in a microfluidic cartridge. The MRPS measurements were performed with an nCS1 instrument (Spectradyne LLC, Torrance, CA, USA). The samples were diluted 500-fold with BSA solution at 1 mg/mL in 0.9% NaCl, filtered through an Amicon Ultra-0.5 centrifugal filter (100 kDa MWCO; Merck KGaA, Darmstadt, Germany), according to the manufacturer’s instructions. All measurements were performed using factory calibrated C-400 cartridges with a measurement range from 65 nm to 400 nm.

Mass spectrometric measurements were performed on a Sciex 6500QTrap (Sciex, Framingham, MA, USA) hybrid quadrupole-ion trap mass spectrometer equipped with Turbo-V ion source. HPLC separations were carried out on an Agilent 1100 system consisting of a binary pump, an autosampler and a column compartment. Analyst 1.6.3. (Sciex, Framingham, MA, USA) software was used for controlling the instrument and for data processing. Quantitation was performed by using the Sciex Multiquant software.

HPLC separation of the samples was performed on a Kinetex EVO C18 (50 × 2.1 mm, 5 μm). The mobile phases were: water containing 0.1% formic acid (eluent A) and acetonitrile containing 0.1% formic acid (eluent B). The separation was performed in gradient elution mode. The initial eluent B composition was 5%. A linear gradient was applied to reach the 95% of eluent B by 5 min and kept there for 1.5 min. The initial solvent composition was set at 0.5 min and the equilibration time was 3 min. The overall run time was 10 min. The flow rate was 0.6 mL/min and the injection volume was 5 µL. The column temperature was ambient.

MS detection in positive ion MRM mode was applied for quantitation of the target molecule. Source parameters were: curtain gas (CUR), nebulizer gas (GS1) and drying gas (GS2) values were set at 40, 40 and 40 arbitrary unit, respectively. The spray voltage was 5500 V. Source temperature was set at 450 °C. Declustering potential was set to 100 V. The PyDau was detected in MRM mode with transitions of 580.2/321 (quantifier) and 580.2/363 (qualifier) (Q1/Q3) with dwell time of 100 ms. Collision energy was 30 eV. A simple sample preparation protocol was applied consisting of a 3x volume of cold acetonitrile for protein precipitation and opening the liposomes. The encapsulation efficiency (EE%) and the drug-to-lipid ratio (D/L) of LiPyDau were calculated according to the following formulae:

1 $${\text{EE}}{\%}=\left({W}_{\rm{entrapped drug}}/{W}_{\rm{initial drug}}\right)\times 100$$

2 $$\:\:\:\text{D}/\text{L}\:=\frac{mol\:drug}{mol\:lipid}$$

where W represents the weight in mg.

Long-term stability of LiPyDau (stored at 4 °C) was assessed using dynamic light scattering (DLS) and spectrophotometric analysis over a one-year period. For drug retention measurements, 200 µL samples were centrifuged through Zeba™ Spin Desalting Columns (40 kDa MWCO, 0.5 mL; Thermo Fisher, Waltham, MA, USA) to remove unencapsulated PyDau, following the manufacturer’s protocol. The liposome-containing fractions were then diluted 1:2 (v/v) with 1% HCl in absolute ethanol to release the encapsulated drug. Absorbance was measured at 485 nm using an EnSpire microplate reader (PerkinElmer, Waltham, MA, USA), and values were normalized to freshly prepared liposomes.

### Cell lines and culture conditions

The NCI-60 cell panel was purchased from NIH, of which 34 cell lines were used in this study, maintained in RPMI medium (melanoma: Malme-3M, SK-MEL-2, SK-MEL-28; colorectal: COLO205, HCC-2998, HCT-116, HCT-15, HT29, KM12, SW-620; lung: EKVX, NCI-H522, NCI-H322M, HOP-62, NCI-H226); prostate: PC-3, DU-145; leukaemia: HL60, CCRF-CEM; ovarian: IGROV-1, OVCAR-3, OVCAR-8, NCI/ADR-RES; gastrointestinal: OVCAR-5; renal: UO-31, A498, CAKI-1, RXF393; breast: BT-549, Hs578T, MDA-MB-231, MCF-7, T-47D, MDA-MB-435. DLD-1 was purchased from ATCC and was maintained in RPMI. Uterine sarcoma cell lines Mes-Sa and Mes-Sa/Dx5 were purchased from ATCC and were cultured in DMEM. Pancreatic cell lines PANC-1 and Bx-PC-3, and lung cancer cell lines NCI-H1792 and LCLC were purchased from ATCC, and were kept in DMEM. Osteosarcoma cell line 143B was a kind gift from Dr. József Balla (University of Debrecen, Hungary), and was maintained in MEM. The media of the above cell lines were supplemented with 10% foetal bovine serum (FBS). DT40 cell lines (described in [17–19]) and gene mutations were verified using whole genome sequence data, and were grown at 37 °C under 5% CO_2_ in RPMI medium supplemented with 7% FBS, 3% chicken serum and 50 µM 2-mercaptoethanol.

### In vitro cytotoxicity studies

Cells were seeded in 40 µl medium on 384 well plates by an automatic liquid handling machine (Hamilton StarLet) at a density of 2500 cells/well. Adherent cells were cultured for 24 h before the addition of the compounds, while drugs were added to non-adherent cells 2 h after cell seeding. The drugs were serially diluted and dispensed in an additional 20 µl. Cells were incubated for an additional 72 h, cytotoxicity was assessed with the PrestoBlue viability reagent (Thermo Fisher). Experiments were repeated at least 3 times. Sigmoidal curve fitting by GraphPad Prism software was applied to determine pIC_50_ values, and the respective IC_50_ values and standard deviations were calculated consequently. The relative IC_50_ values calculated as log10(IC50_mutant_)-log10(IC50_WT_) were clustered using the *superheat* function in R.

### Animal experiments

Animal experimental procedures were approved and performed in accordance with the guidelines of the Institutional Animal Care and Use Committee at the National Institute of Oncology (Budapest, Hungary) under Animal housing density according to the regulations and recommendations from directive 2010/63/EU of the European Parliament and the Council of the European Union on the protection of animals used for scientific purposes. Permission license for breeding and performing experiments with laboratory animals: PEI/001/1738-3/2015 and PE/EA/1461-7/2020. The animals used in this study were cared for according to the “Guiding Principles for the Care and Use of Animals” based upon the Helsinki declaration, and they were approved by the local ethical committee. The mice were kept in a sterile environment in Makrolon^®^ cages at 22–24 °C (40–50% humidity), with a lighting regulation of 12/12 h light/dark. The animals had free access to water and were fed with a sterilized standard diet (VRF1, autoclavable, Akronom Kft., Budapest, Hungary) *ad libitum*.

### In vivo toxicity studies

PyDau and LiPyDau dose-finding studies were performed using 10-week-old FVB mice, by single intravenous injections of different doses of the compounds (3 mice/dose) via the tail vein. The status of the mice was monitored daily for signs of pain or discomfort. Mice were euthanized at 20% body weight loss or at a low score (1 or 2) on the Body Condition Scoring scale. Survival was followed for 30 days and the Maximum Tolerable Dose (MTD) was defined as the highest dose where 100% survival was observed.

### Xenograft models

Xenograft tumors were established from the uterine sarcoma cell lines MES-SA and its multidrug resistant derivative MES-SA/Dx5. 2 × 10^6^ cells from each cell line in 200 µl serum-free culture media DMEM were inoculated into NOD.Cg-Prkdcscid Il2rgtm1Wjl/SzJ (NSG) mice subcutaneously in the left flank and treatment was initiated when tumors reached 200 mm^3^. The size of the tumors was measured every second day with a digital caliper.

### Human patient-derived tumor xenograft in mice

An 83-year-old patient was diagnosed with invasive lung adenocarcinoma in 2016 following the surgical removal of a brain metastasis, and its histological characterization at the 2nd Department of Pathology, Semmelweis University, Budapest, Hungary. Five months later, the primary pulmonary tumor was removed at the National Korányi Institute of Pulmonology, Budapest, Hungary, and sequencing revealed wild type EGFR (exons 18–21), KRAS (codons 12 & 13), with no structural change of ALK gene, rendering the patient ineligible for available targeted therapy. The establishment of the xenograft was done as described previously [20]. Briefly, a 4–10 mm^3^ piece from the tumor tissue was transplanted subcutaneously into the right flank and propagated in three 8-week-old male NOD.Cg-Prkdcscid Il2rgtm1Wjl/SzJ (NSG) mice for 7 months. After the xenografted tumor reached 1000 mm^3^, tumor pieces were retransplanted subcutaneously into the right flank of 20 additional NSG mice, which were given a single treatment with various chemotherapeutics or LiPyDau. After daily monitoring of the tumor volume for one week, the mice were sacrificed and the cytotoxicity of the treatments was investigated on tissue slices by image analysis.

### Institutional review board statement

KINETO Lab Kft. has license for animal housing (PEI/001/1715/2015) and PDTX sample collection, handling, and model generation and use (IV/10147-1/2020/EKU). The provided PDTX samples were transferred on dry ice to the experimental animal house of the National Institute of Oncology, Budapest, Hungary. All ethical permissions were given by the Scientific and Research Ethics Committee, a national board in Hungary. Mouse euthanasia was carried out using overanesthesia by 1% isoflurane inhalation, followed by cervical dislocation.

### Mouse allograft models

Mouse melanoma (B16-F1 subline exhibiting low metastatic potential [21]), lung (Lewis lung carcinoma, LLC) and breast (4T1-LUC) cancer cell lines were used to establish allograft tumors for drug testing. 5 × 10^5^ B16 or 2 × 10^5^ LLC cells were inoculated subcutaneously in 200 µl serum-free culture media. 4T1-LUC tumors were established by injecting 1 × 10^6^ cells in 20 µl serum-free culture media through the nipple directly to the breast tissue of 8 weeks old BALB/c mice on day 0. Growth of 4T1-LUC tumor was monitored by bioluminescence imaging. Images were taken 10 min after tail vein injection of 150 mg/kg dose of D-luciferin into the mouse using an IVIS Lumina III in vivo imaging system (PerkinElmer, Waltham, Massachusetts). The in vivo imaging experiments were performed in the Small Animal Imaging Facility at the University of Pécs Medical School, Department of Pharmacology and Pharmacotherapy belonging to the Euro Bioimaging Medical and Preclinical node Hungary. The experiments with the 4T1-LUC model were performed under the license BA02/2000-9/2019.

### Genetically engineered mouse model of triple-negative breast cancer

Tissue pieces (1–2 mm in diameter) obtained from Brca1^−/−^;p53^−/−^ FVB mouse mammary tumors (a kind gift from Sven Rottenberg, NKI) were transplanted orthotopically into the mammary fat pad of wild type FVB/NHanHsd mice (Harlan, Italy) under anesthesia (20 mg/kg zolazepam, 12.5 mg/kg xylazine, 3 mg/kg butorphanol, 20 mg/kg tiletamine). Organoid lines were transplanted as described [22]. Briefly, organoids were used at a size corresponding to an average of 150–200 cells per organoid. Organoid suspensions containing a total of 5 × 10^4^ cells in 30 µl of organoid media/BME mixture (1:1) were injected orthotopically into the fourth right mammary fat pad of 7-week-old wild-type FVB/N mice (Charles River Laboratories, Germany) under anesthesia (100 mg/kg ketamine and 5 mg/kg xylazine) after a small surgical incision. The tumor size was monitored at least 3 times per week by caliper measurements after the tumors became palpable. Tumor volume was calculated using the V = length× (width^2^)/2 formula. When the volume of the tumors reached ~ 200 mm^3^, DOX treatment was initiated using the maximum tolerable dose (MTD, 5 mg/kg iv). Treatments using the MTD of DOX were repeated every 10 days unless the size of the tumors decreased to 50% of its original volume. In that case treatment was repeated when the tumor relapsed to its original size. The FEC protocol (24 mg/kg fluorouracil, 4 mg/kg epirubicin and 120 mg/kg cyclophosphamide) was administered every 21 days for a maximum of 6 cycles. Animals were sacrificed when the tumor volume reached ~ 2000 mm^3^.

### Image analysis

Hematoxylin and eosin-stained tissue sample images were acquired using a Pannoramic 1000 whole-slide scanner at 20× objective magnification and a resolution of 0.25 μm/pixel. The digitized images were then downscaled by a factor of 16 to reduce their size for automated processing. At this resolution, individual cells are no longer visible, but distinct tissue regions remain identifiable. To quantify the proportion of necrotic tissue segments, a two-step automated image processing approach was applied. First, a pixel classification was performed on the resized sample images using Ilastik [23]. Each pixel was assigned one of three predefined labels (viable tissue, necrotic tissue, background) based on a random decision forest classifier trained on a set of histologist-annotated samples. The classifier utilized color, intensity, edge, and texture features of the input images to predict labels. The resulting probability maps were then processed using a custom-built CellProfiler pipeline [24]. The classification acceptance threshold for each category was set to 50%. As the resulting segmentation of the original images often contained small artifacts, median filtering using a 5 × 5 sampling window was utilized to remove the noise from the images. Finally, for each image, a necrotic tissue ratio $\:{\eta\:}_{I}$ was calculated as

$${\eta}_{I}=\frac{{N}_{I}}{{N}_{I}+{V}_{I}},$$

where $\:{N}_{I}$ and $\:{V}_{I}$ denote the number of pixels on the given image classified as necrotic and viable, respectively. The necrotic tissue ratio ranges from 0 to 1. A value of 0 indicates that no part of the original slide was classified as necrotic tissue, whereas a value of 1 signifies complete tissue destruction. Thus, $\:\eta\:$ serves as a measure of treatment efficacy.

### Helicase and binding studies

The fluorescent 5’-Rhodamine

G-GTCTGATCGTTTTCCGTTGCTCTGGTTAAGTCGTTTTTTTTTTTTTTTTTTTTTT-3’

(Fluo34T21) and the quencher bearing

5’-ACGACTTAACCAGAGCAACGGAAAACGATCAGAC-IOWA BLACK3’ (Q34)

oligos were synthesized and HPLC purified by IDT. The two oligos were annealed at equimolar concentrations and forming 34 bp double stranded part with a 21 bp 3’ poly (T) overhang that ensures size discrimination between the two strands and served for the binding of BLM helicase in helicase studies. The long (2880 bp) dsDNA was obtained by opening a pUCHSO derivative plasmid with EcoRI and purified from agarose gel.

Inhibition of BLM helicase driven real-time strand separation by Dau and PyDau was measured fluorometrically. A capture oligo (5’-GTCTGATCGTTTTCCGTTGCTCTGGTTAAGTCGT-3’) complementary to the Q34 oligo was applied here at 20-fod excess and served for capturing the quencher containing oligo (base-pairing with it) after the helicase has separated it from the Fluo34T21 oligo. The liberated fluorescence was measured by a Cary Eclipse spectrofluorometer equipped with a Peltier four-position multicell holder accessory and a temperature controller (Varian) using excitation/emission wavelength of 504/531 nm, respectively. Reactions were performed in 1.0 ml cuvettes, in a buffer of 10 mM magnesium acetate, 10 mM Tris, 40 mM potassium acetate, (pH 7.4). First the reagents DAU and PyDau at 0.1 and 1 µM concentrations (reagent excess/bp of DNA was 1.4/0.14, respectively) were preincubated with the DNA (21 nM) for 3 h in assay buffer at 37 °C, then the individual mixtures were supplemented with a concentrated master mix containing the essential components for the helicase reactions to attain the final concentration of 2 mM ATP, 10 mM phosphocreatine, 2.5 U creatinine phosphokinase, 0.4 mM DTT, 0.2 µM BSA and 0.42 µM capture oligo (20-fold excess) concentration. BLM _(642−1191)_ helicase was added to the reaction to final concentration of 15.4 nM after starting the measurement and obtaining a stable baseline. Measurements were done at 25 °C. The data were analysed using the instrument software fitting the data to the first-order rate equation. A perfect fit was observed. All experiments were executed in three repeats including the mock control (without reagent).

In binding studies dsDNAs of two various sizes, UREA polyacrylamide and alkaline agarose gel electrophoresis was applied. The shortest (34 bp) fluorescent dsDNA construct (used also in helicase assays) obtained by annealing Fluo34T21 and Q34 was incubated in a 10 µl volume without any reagent (mock control) or with DAU and PyDau at 1.0 µM DNA concentration (14.7-fold reagent excess/bp of DNA) at 37 °C for 300 min in 1X helicase buffer (10 mM Magnesium acetate, 10 mM Tris, 40 mM Potassium acetate, pH 7.4). Two volumes of loading buffer containing 10 M Urea, 10 mM EDTA, 5 mM TRIS (pH 7.5), 0,5% bromophenol blue, 10% glycerol was then added, and the samples were heat-treated at 95 ℃ for 2 min, then applied to a 10% Mini-PROTEAN TBE-Urea precast PA gel (BioRad) that was run at 55 ℃ at 30 V in 1X TBE buffer. At the end of the run the gel was stained with 3000X GelRed stain in 1X TBE buffer, washed in 1X TBE and scanned at two channels (GelRed and Fluorescein epi-green). Images were processed by the Image Lab 4.1 software.

The plasmid-sized DNA (2880 bp at 0.013 µM) was also incubated with DAU, DOX and PyDau at 0.006 reagent excesses for 300 min followed with the removal of the excess reagents by phenol-chloroform purification and with alkaline agarose gel electrophoresis.

### Molecular docking

Covalent docking calculations were performed with the CovDock module [25] of the Schrödinger Small-Molecule Drug Discovery Suite 2025-2 [Schrödinger Release 2025-2: Glide, CovDock, Schrödinger, LLC, New York, NY, 2025.], using the formaldehyde cross-linked daunorubicin containing PDB structure (1D33) as receptor [26]. During receptor preparation, missing backbone atoms of the PDB structure were built in manually. Docking grids were generated using the default options. During the CovDock calculations 3 docking poses were generated with the thorough pose prediction setting. Binding pose metadynamics simulations [27] were performed on the two best ranking predicted complexes to test their stability using the Desmond program [Schrödinger Release 2025-2: Desmond Molecular Dynamics System, D. E. Shaw Research, New York, NY, 2024. Maestro-Desmond Interoperability Tools, Schrödinger, New York, NY, 2025]. Figures were generated using the Maestro program [Schrödinger Release 2025-2: Maestro, Schrödinger, LLC, New York, NY, 2025].

## Results

### 2-pyrrolino-daunorubicin is highly toxic against a diverse cancer cell line panel including multidrug resistant cell lines

**Fig. 1:**
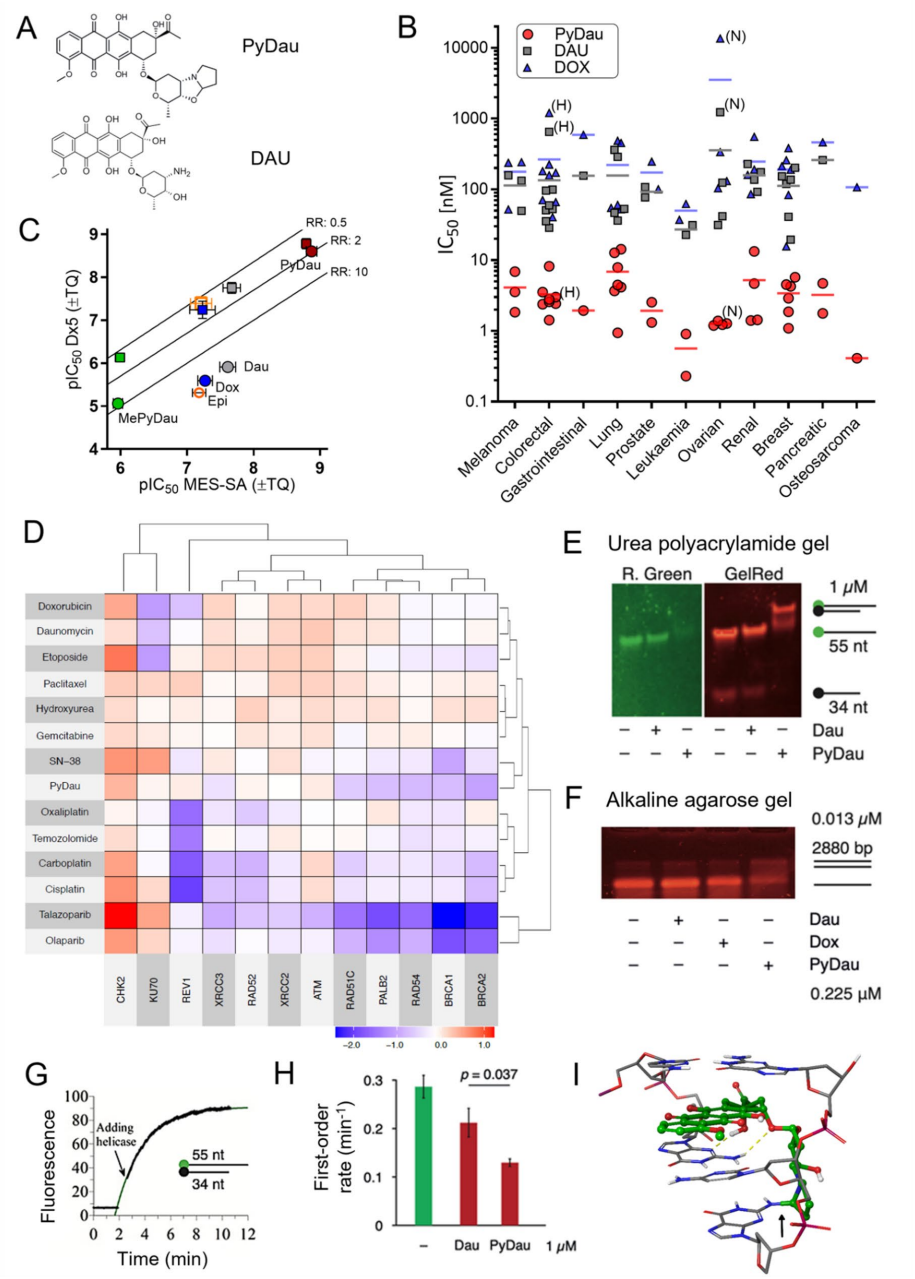
PyDau is up to 1000 times more toxic than DAU or DOX and shows a distinct activity pattern across multiple cell panels, linked to interstrand DNA crosslinks that lead to irreparable DNA damage. **A** Structures of PyDau and DAU. **B** IC_50_ values of PyDau (red circles) measured in 40 human cancer cell lines of the indicated tissue of origin (see Table S1 for details). Each data point represents the IC_50_ of an individual cell line, horizontal lines indicate the average IC_50_ within tissue groups. P-gp expressing MDR lines HCT-15 and NCI/ADR-RES cells are indicated with (H) and (N), respectively. IC_50_ values of DOX (blue triangles) and DAU (gray squares) were taken from the NCI-60 Screening Database [31] and from Varbanov et al. [32] for the value measured in PANC-1 cells. **C** Cytotoxicity of anthracycline analogs in parental MES-SA and multidrug resistant MES-SA/Dx5 cells, displayed as pIC_50_ values, tested alone (circles) or in the presence of the ABCB1/P-gp inhibitor tariquidar (squares). While PyDau (red) exhibits equal toxicity to parental and MDR cells, the toxicity of MePyDau (green), epirubicin (orange), DOX (blue), DAU (gray) is significantly reduced in multidrug resistant MES-SA/Dx5 cells. This reduced toxicity is reversed in the presence of the ABCB1/P-gp inhibitor tariquidar. RR: resistance ratio, IC_50_MDR_/IC_50_parental_. **D** Clustering of the tested compounds based on relative pIC_50_ values measured against a panel of DT40 cell lines missing the indicated DNA repair genes. Red color refers to relative resistance of a cell line, while blue color refers to relative hypersensitivity, as compared to the sensitivity of WT cells. A subset of the data was published earlier [18]. **E** A double-stranded oligonucleotide with a green fluorophore attached to the 55 nt strand, and a quencher attached to the 34 nt strand was incubated with 500 µM DAU or PyDau (14.7-fold reagent excess/base pair of DNA) for 180 min. The double-stranded oligos were then run on a 8 M urea polyacrylamide gel, stained with GelRed, and visualized in two channels (GelRed and Fluorescein epi-green). In the control and DAU-treated samples, separation of the two strands results in the appearance of a 55 nt band showing green fluorescence. A low mobility band is observed only after PyDau treatment, indicating non-separable (cross-linked) DNA with quenched green fluorescence. **F** A 2880 bp dsDNA fragment was incubated with 0.225 µM DAU, DOX or PyDau (at 0.006 reagent molecules/base pair of DNA) and separated on an alkaline agarose gel. The low mobility band, indicating non-separable (cross-linked) DNA, is formed even at a very low PyDau excess. **G** Fluorometric helicase activity assays using a dsDNA substrate with fluorophore and quencher labels as in (E), were initiated by the addition of the 642–1191 fragment of human BLM helicase at a concentration of 15.4 nM. Helicase reactions followed first-order kinetics, as indicated by the fitted curve. **H** Inhibition of strand separation by DAU or PyDau represented by the reduction in first-order rate constants. The mean and S.E.M. of three measurements are shown, with the indicated significance value derived from an unpaired t-test. **I** Top-ranking conformation of the covalently docked PyDau–DNA complex. DNA is shown in stick representation with gray-colored carbon atoms, while PyDau is shown in ball-and-stick representation with green-colored carbon atoms. Yellow dotted lines indicate hydrogen bonds; the arrow points at the covalent bond between PyDau and the DNA strand. See Supplementary Figure S3 for more details

2-pyrrolino-daunorubicin (PyDau, Fig. 1A) was synthesized by a method minimizing the formation of side products (Figure S1). As compared to DAU, PyDau was up to 1000-fold more toxic against a diverse panel of cancer cell lines, with IC_50_ values in the nanomolar range (Fig. 1B, Table S1). While the toxicity of DOX and DAU is significantly decreased by P-glycoprotein (P-gp), PyDau was able to overcome the resistance of NCI/ADR-RES, HCT-15 and Mes-Sa/Dx5 expressing high levels of P-gp (Fig. 1B and C). The toxicity of DAU is associated with its ability to act as a topoisomerase II inhibitor, the formation of interstrand DNA cross-links and the generation of free radicals [28, 29] while the mechanism of action of PyDau is not known. High toxicity of a related compound, PyDox was attributed to the alkylating function of the daunosamine nitrogen incorporated into the pyrrolino ring [30]. In line with this mechanism of action, α-methyl-2-pyrrolino-daunorubicin (MePyDau, Figure S2), in which the pyrrolino ring cannot react with DNA, was found to be 3 orders of magnitude less toxic than the parental compound (Fig. 1C). To characterize the role of DNA interactions in the mechanism of toxicity of PyDau, we measured its activity pattern across a panel of DT40 chicken lymphoblast cell lines missing individual components of DNA-repair enzymes (Fig. 1D). As expected, deletion of key DNA repair enzymes sensitized DT40 cells to most chemotherapeutic drugs (Table S2). While PyDau was significantly more toxic than the other characterized chemotherapeutics, its activity pattern across the single-KO DT40 cells was more similar to the profile of the topoisomerase I inhibitor SN-38 than to that of the parent compound DAU or DOX. Hypersensitivity of homologous recombination (HR) deficient cells and the shared activity pattern with DNA double strand break-inducing agents suggest that PyDau has a distinct mechanism as compared to the parental anthracyclines. Indeed, PyDau effectively induced interstrand DNA crosslinks in short and long dsDNA molecules withstanding 8 M urea or alkaline denaturation and inhibiting strand separation by a DNA helicase more effectively than DAU (Fig. 1E-H). Molecular docking simulations confirmed that PyDau is capable of forming a covalent bond with one DNA strand and hydrogen bonds with the complementary strand, offering a plausible explanation for the observed crosslinking results (Fig. 1I, Figure S3-4). Taken together, these results suggest that the extremely strong cytotoxicity of PyDau is mediated by its strong binding to DNA through the reactive pyrrolino-group and the intercalation between base pairs, akin to the formation of covalent adducts formed by anthracyclines in the presence of formaldehyde [28, 30]. The resulting crosslinking of DNA strands leads to double strand breaks, and ultimately to cell death.

### Liposomal formulation enhances the therapeutic window of PyDau to allow efficient anticancer treatments

**Fig. 2:**
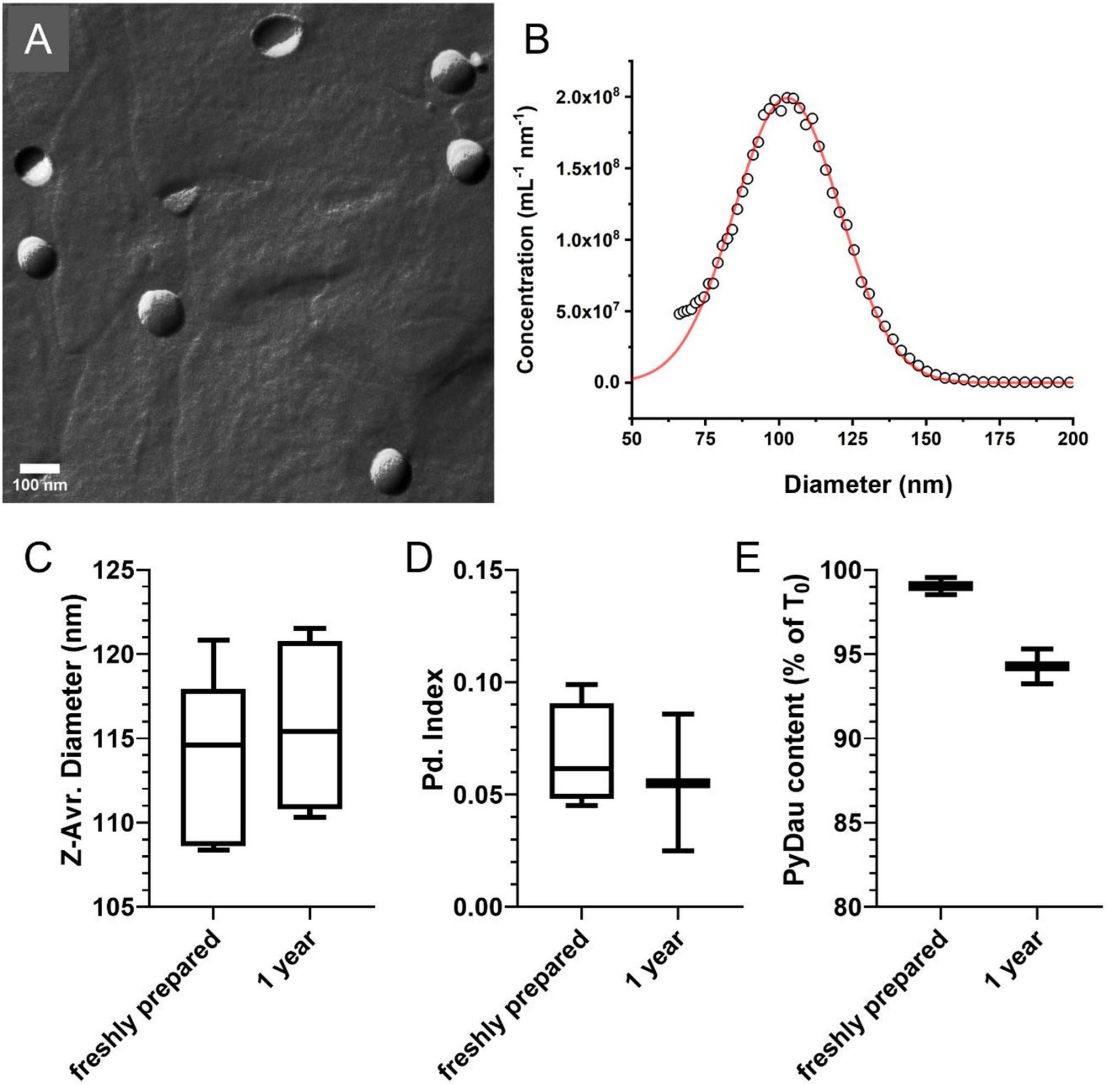
**Characterization of PyDau loaded liposomes (LiPyDau). A** Freeze-fracture combined transmission electron microscopy (FF-TEM) image of PyDau-loaded liposomes (LiPyDau). **B** Absolute size distribution of LiPyDau measured by microfluidic resistive pulse sensing (MRPS). **C**-**E** Long-term stability of LiPyDau liposomes over one year. **C** Average hydrodynamic diameter; **D** polydispersity index (PDI); and **E** encapsulated PyDau content of freshly prepared and one-year stored LiPyDau samples. Size and PDI were determined by dynamic light scattering (DLS); PyDau content was assessed spectrophotometrically

Preliminary dose-finding studies indicated that PyDau exerts significant in vivo antitumor effect, which is, however, limited by the rapidly accumulating toxicity associated with the treatment (Table S3). Based on our previous work characterizing the effect of PEGylated liposomal DOX on multidrug-resistant mammary tumors [41], we produced PyDau-loaded PEGylated liposomes (LiPyDau) using the lipid film hydration and extrusion method [33–35]. PyDau was loaded into the liposomes using the ammonium-sulfate gradient technique with 78 ± 12% enapsulation efficiency corresponding to a drug-to-lipid ratio (D/L ratio) of 0.20 ± 0.05. LiPyDau possesses spherical morphology characteristic of unilamellar liposomes and monodisperse size distribution with an average diameter of 102.8 nm and full width at half maximum of 43.6 nm (Fig. 2A and B), as revealed by freeze-fracture combined transmission electron microscopy (FF-TEM) and microfluidic resistive pulse sensing (MRPS). According to in vitro stability tests, LiPyDau maintained its liposomal structure retaining 95% of the encapsulated PyDau for at least a year (Fig. 2C-E).

**Fig. 3:**
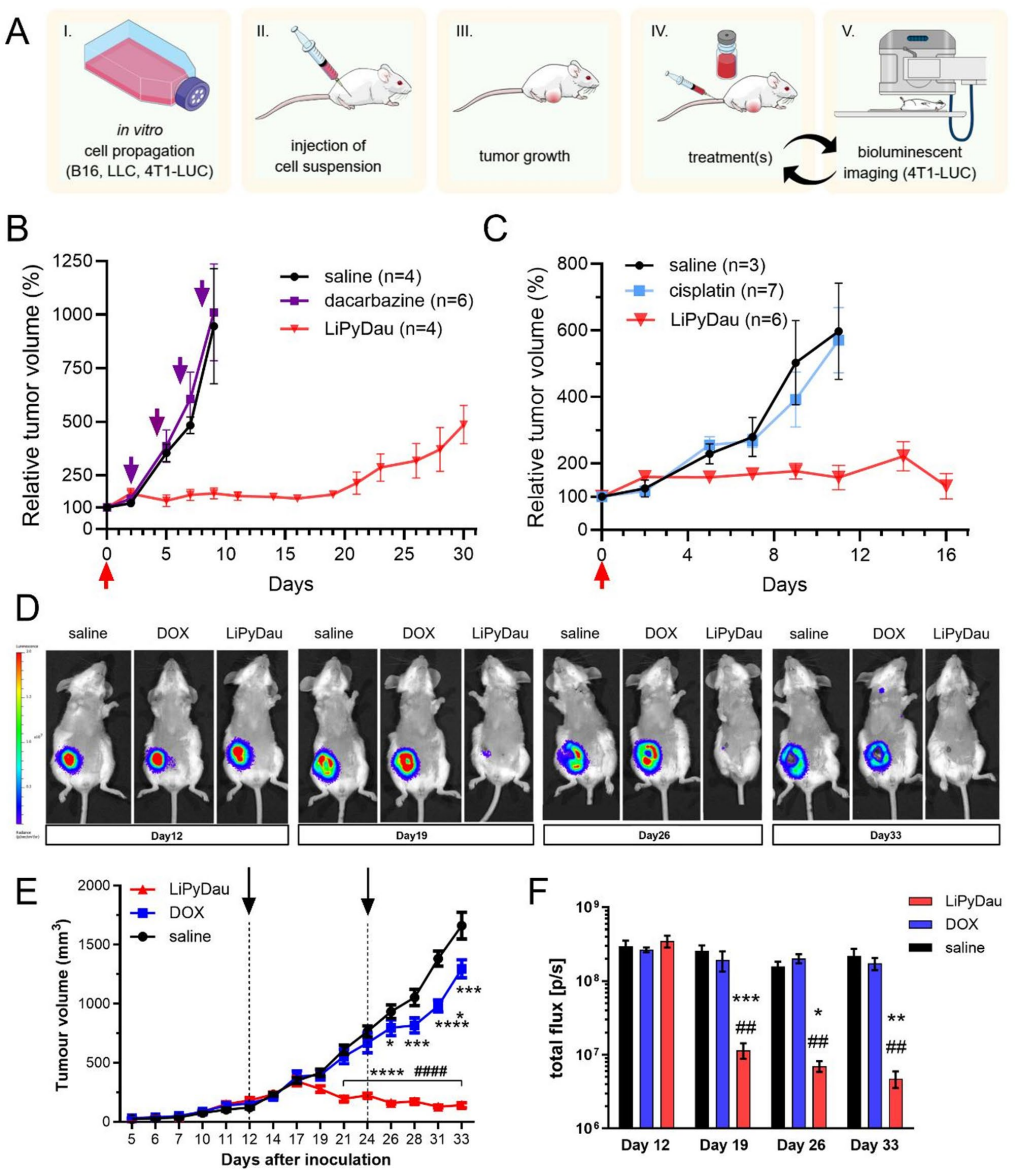
Significant and long-lasting anticancer effect of LiPyDau across allograft mouse cancer models. **A** Schematic representation of the allograft experiments. Key steps include in vitro culturing of cell lines, tumor cell implantation, treatment schedule (e.g., single-dose administration of LiPyDau, DOX, or control), and the timeline of tumor monitoring and sample collection. **B** Tumor growth inhibition in the B16 melanoma allograft model following treatment with LiPyDau. Mice were subcutaneously engrafted with 5 × 10^5^ B16 cells; treatment with dacarbazine (iv 70 mg/kg, indicated by downward arrows, *n* = 6), saline (*n* = 4) or LiPyDau (iv 1 mg/kg, single treatment, upward arrow, *n* = 4) was initiated when the tumors reached 100 mm^3^. **C** Tumor growth inhibition in the Lewis lung carcinoma (LLC) allograft model. LLC tumor cells (2 × 10^5^) in 100 µl phosphate-buffered saline were subcutaneously inoculated into the right flanks of C57BL/6 mice. After 12–14 days, mice with palpable tumors were randomized and treated with a single intravenous dose of cisplatin (5 mg/kg), LiPyDau (1 mg/kg) or saline, as indicated by the arrow. **D** Tumor growth inhibition in the 4T1 breast cancer allograft model. Representative whole animal bioluminescence images corresponding to the indicated time points. Orthotopic breast tumors were established by injecting 1 × 10^6^ 4T1-LUC cells in 20 µl serum-free culture media through the nipple directly to the breast tissue of 8-week-old BALB/c mice on day 0. Mice were randomly assigned to groups to receive intravenous treatment with either DOX (5 mg/kg, *n* = 6), LiPyDau (1 mg/kg, *n* = 6) or saline (*n* = 6) on days 12 and 24. **E** Tumor volume measurements corresponding to the experiment shown in Panel D. Tumor growth was monitored by caliper measurements over time to assess treatment efficacy. Arrows indicate treatments. Statistical analysis was conducted using two-way ANOVA followed by Tukey’s multiple comparison test (* *p* < 0.05, **** *p* < 0.0001 vs. saline; <0.0001 vs. DOX). F. Quantification of total photon flux (photons/second) at tumor regions shown in panel D. Statistical analysis was performed with two-way ANOVA and Tukey’s multiple comparison test (* *p* < 0.05, ** *p* < 0.01, vs. saline; ## *p* < 0.01 vs. DOX)

In vivo dose-finding studies confirmed that LiPyDau is tolerated at doses reaching 1.5 mg/kg, without any signs of toxic side effects (Table S4). Next, we tested the therapeutic potential of LiPyDau in several preclinical allograft and xenograft mouse models (Fig. 3A). LiPyDau efficacy was first evaluated in mouse allograft tumors using melanoma (B16), lung (Lewis lung carcinoma, LLC) and breast (4T1-LUC) cancer cell lines, comparing the antitumor effect of intravenous LiPyDau to first-line chemotherapy drugs used in the treatment of the given cancer type. LiPyDau exhibited significant and long-lasting anticancer activity across all models. As shown in Fig. 3B, repeated treatments with dacarbazine, the only single-agent approved by the Food and Drug Administration for treating metastatic melanoma [36], did not exhibit any effect on the growth of B16 melanoma tumors. In contrast, treatment with a single dose of LiPyDau resulted in an almost complete inhibition of tumor growth for at least 20 days. Similarly, whereas treatment with cisplatin did not have any measurable effect on the growth of LLC tumors, a single dose of LiPyDau resulted in a significant antitumor effect (Fig. 3C). Inoculation of luciferase-expressing 4T1 (4T1-LUC) cells in the mammary fat pad of female BALB/c mice results in the formation of highly malignant and metastatic breast tumors, allowing continuous monitoring of the location, growth and viability of tumor cells by bioluminescence imaging. As shown in Fig. 3D-F, repeated treatment with the MTD (5 mg/kg) of doxorubicin had negligible effects. In contrast, two cycles of 1 mg/kg LiPyDau treatment suppressed tumor growth, leading to almost complete tumor cell elimination and a prolonged antitumor effect up to the experimental endpoint at day 33. This was accompanied by notable but non-life-threatening weight loss (Figure S5) and palmar-plantar erythrodysesthesia, a characteristic side effect of pegylated liposomes [37].

**Fig. 4:**
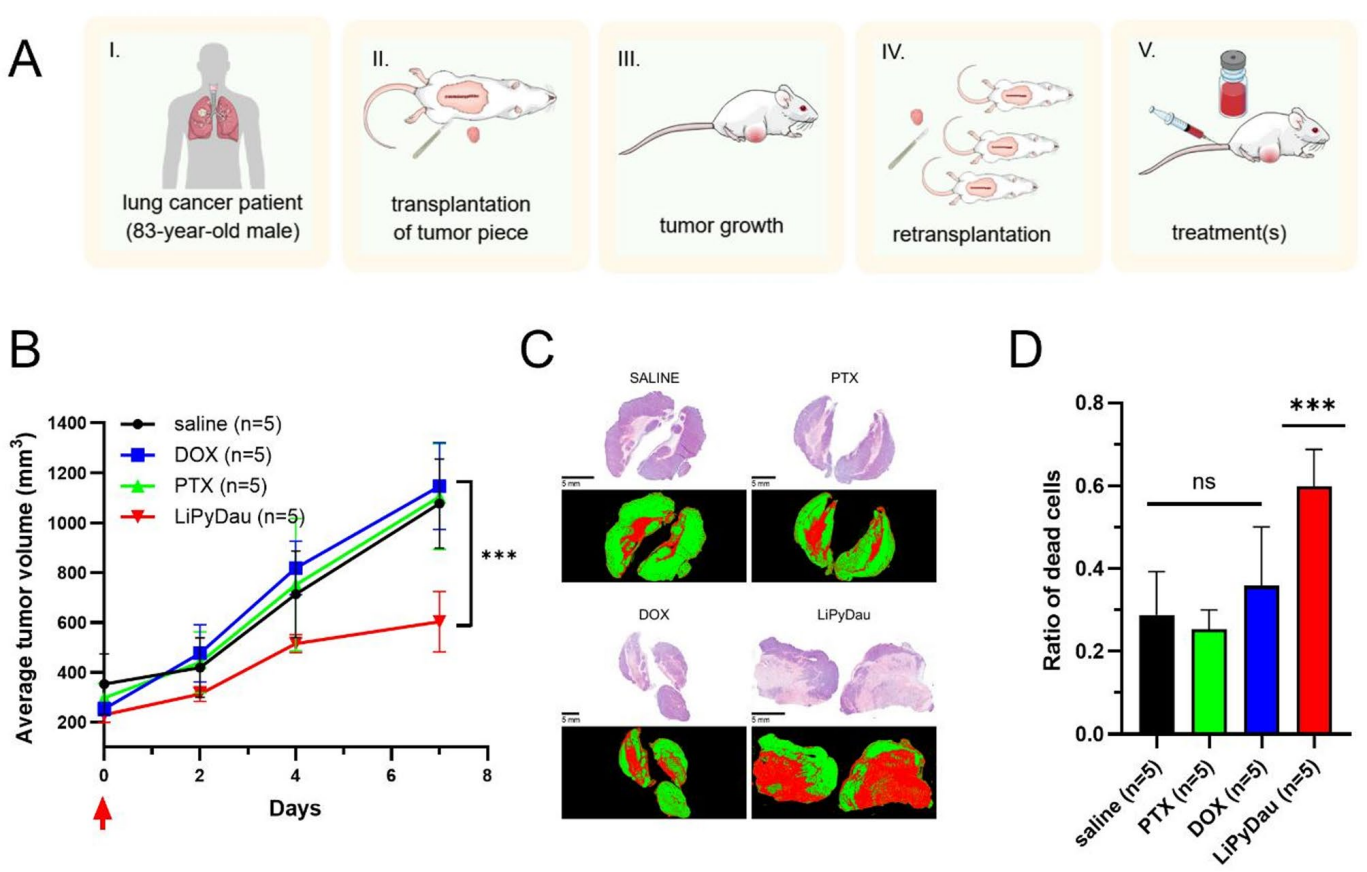
LiPyDau treatment inhibits the growth of a patient-derived lung adenocarcinoma xenograft. **A** Schematic representation of the patient-derived tumor xenograft experiments. The diagram outlines key steps of the xenograft model: tumor biopsy from a human patient, subcutaneous implantation of tumor cells into immunodeficient mice, tumor growth and stabilization, re-transplantation to expand the model, treatment administration, and the experimental timeline for tumor volume measurements and survival monitoring. **B** Tumor growth of a lung adenocarcinoma PDTX model intravenously treated with saline, DOX (2 mg/kg, *n* = 5), paclitaxel (PTX; 12 mg/kg, *n* = 5), or LiPyDau (0.5 mg/kg, *n* = 5) at day 0, indicated by the arrow. Statistical analysis was conducted using Tukey’s multiple comparison test. *** *p* < 0.001 **C** High resolution images of hematoxylin and eosin-stained tumor sections (deep blue-purple images) and the result of the automated image processing used to determine the ratio of viable (green) and necrotic (red) tissue segments. Scale bars were set to 5 mm. **D** Ratio of the surface area corresponding to dead cells in the different treatment groups. Statistical analysis was conducted using ordinary one-way ANOVA test. *** *p* < 0.001

Next, LiPyDau efficacy was evaluated against a patient-derived tumor xenograft (PDTX) established from an 83-year-old human patient diagnosed with invasive lung adenocarcinoma. Sequencing of the tumor revealed the lack of EGFR, KRAS or ALK mutations, rendering the patient ineligible for targeted therapy options. For treatment, early passage tumor pieces were engrafted subcutaneously into NSG mice, and mice were randomized to receive the indicated therapies when the tumor volume reached ~ 200 mm^3^ (Fig. 4). Unlike DOX or paclitaxel, a single dose of LiPyDau significantly suppressed the growth of patient-derived xenograft adenocarcinoma tumors. Histopathological evaluation of drug-treated tumors excised from mice immediately after the end of treatment showed significant cell death resulting from LiPyDau treatment.

**Fig. 5:**
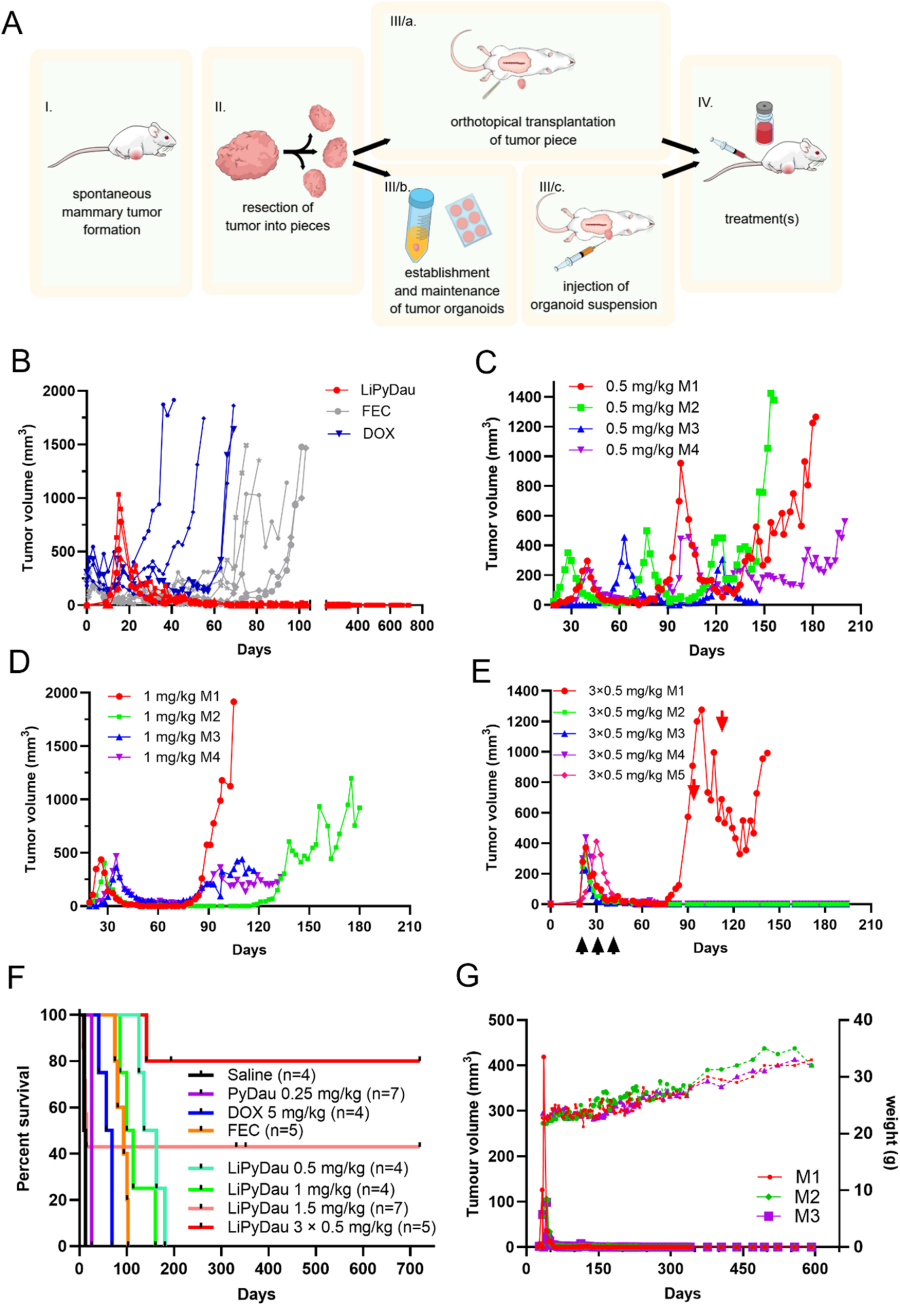
LiPyDau treatment eradicates mammary tumors in a genetically engineered mouse model of TNBC. **A** Schematic representation of the GEMM experiments. The diagram outlines key steps of the GEM model: tumor fragments are collected from spontaneously arising mammary tumors in the KBP1 GEM model and orthotopically engrafted into wild-type recipient mice. Alternatively, tumor-derived organoids are established and subsequently engrafted into wild-type mice. Following tumor establishment and stabilization, treatment is administered, and tumor volume and survival are monitored over the experimental timeline. **B** Mice were intravenously treated with 5 mg/kg DOX (blue), or the FEC protocol (gray), or 1.5 mg/kg LiPyDau (red). Growth kinetics of individual Brca1^−/−^;p53^−/−^ tumors engrafted into the mammary fat pad of wild-type female FVB mice. Treatments were administered when the tumors reached 200 mm^3^. **C** Treatment with 0.5 mg/kg LiPyDau. **D** Treatment with 1 mg/kg LiPyDau. **E** LiPyDau treatment at a cumulative dose of 1.5 mg/kg, administered over a period of 21 days (0.5 mg/kg/week). **F** Overall survival of treatment groups B-E. **G** Organoid-derived tumors were treated with a single dose of 1.25 mg/kg LiPyDau (continuous lines). Dashed lines represent the weight change of the mice

The therapeutic potential of LiPyDau was further tested in the KB1P murine model of hereditary triple negative breast cancer (TNBC) [38]. Conditional deletion of Brca1 and p53 in epithelial cells results in the formation of murine mammary tumors closely mimicking TNBC in human patients [39]. Orthotopically transplanted Brca1^−/−^;p53^−/−^ tumors into the mammary fat pad of wild-type mice offers a simple means to study response to treatment (Fig. 5A). Like most human cancers, Brca^−/−^;p53^−/−^ tumors show initial sensitivity to DOX and to combination treatments such as FEC (5-fluorouracil, epirubicin and cyclophosphamide). However, resistance is eventually acquired after multiple treatment cycles (Fig. 5B). In contrast, treatment with a single, maximum tolerated dose (MTD) of 1.5 mg/kg LiPyDau resulted in a significant and long-lasting antitumor response, extending up to 720 days (Fig. 5A). Although this regimen, using the MTD established in short term experiments, was curative, dose-limiting toxic side effects were observed in 4/7 mice. To further optimize treatment, alternative dosing regimens were tested. Mice received either three doses of LiPyDau at 0.5 mg/kg (Fig. 5C) or two doses of 1 mg/kg (Fig. 5D), administered at each tumor relapse when the relapsing tumors reached the same size as during the initial treatment. Both tumor-triggered protocols, which resulted in cumulative doses of 1.5 mg/kg and 2 mg/kg, respectively, produced significant but not curative antitumor effects. However, when treatment was administered on a fixed schedule—fractionated into three separate injections of 0.5 mg/kg over 21 days—tumors were eradicated in all but one case (Fig. 5E). The overall survival of the treatment groups is shown in Fig. 5F. Compared to the survival times achieved with treatment of 6 cycles of DOX (up to 69 days), 6 cycles of FEC (up to 103 days), LiPyDau treatment fractionated over 21 days at a cumulative dose of 1.5 mg/kg provided a substantially prolonged survival benefit, effectively curing the mice. Finally, organoids derived from KB1P tumors [22] were engrafted into the 4th mammary fat pad of syngeneic FVB/N female mice. Mice developing mammary tumors (~ 100 ± 25 mm^3^) within 50 days following inoculation (3/8) were treated with LiPyDau. A single dose treatment with 1.25 mg/kg LiPyDau completely eradicated the organoid-derived tumors without any sign of toxicity (Fig. 5G). Mice were sacrificed 600 days after tumor engraftment, and the liver, kidneys, spleen, heart, lungs, brain were subjected to histopathological analysis. No major alterations were observed in any of the tissues (Figure S6-7), indicating the long-term safety of LiPyDau.

**Fig. 6:**
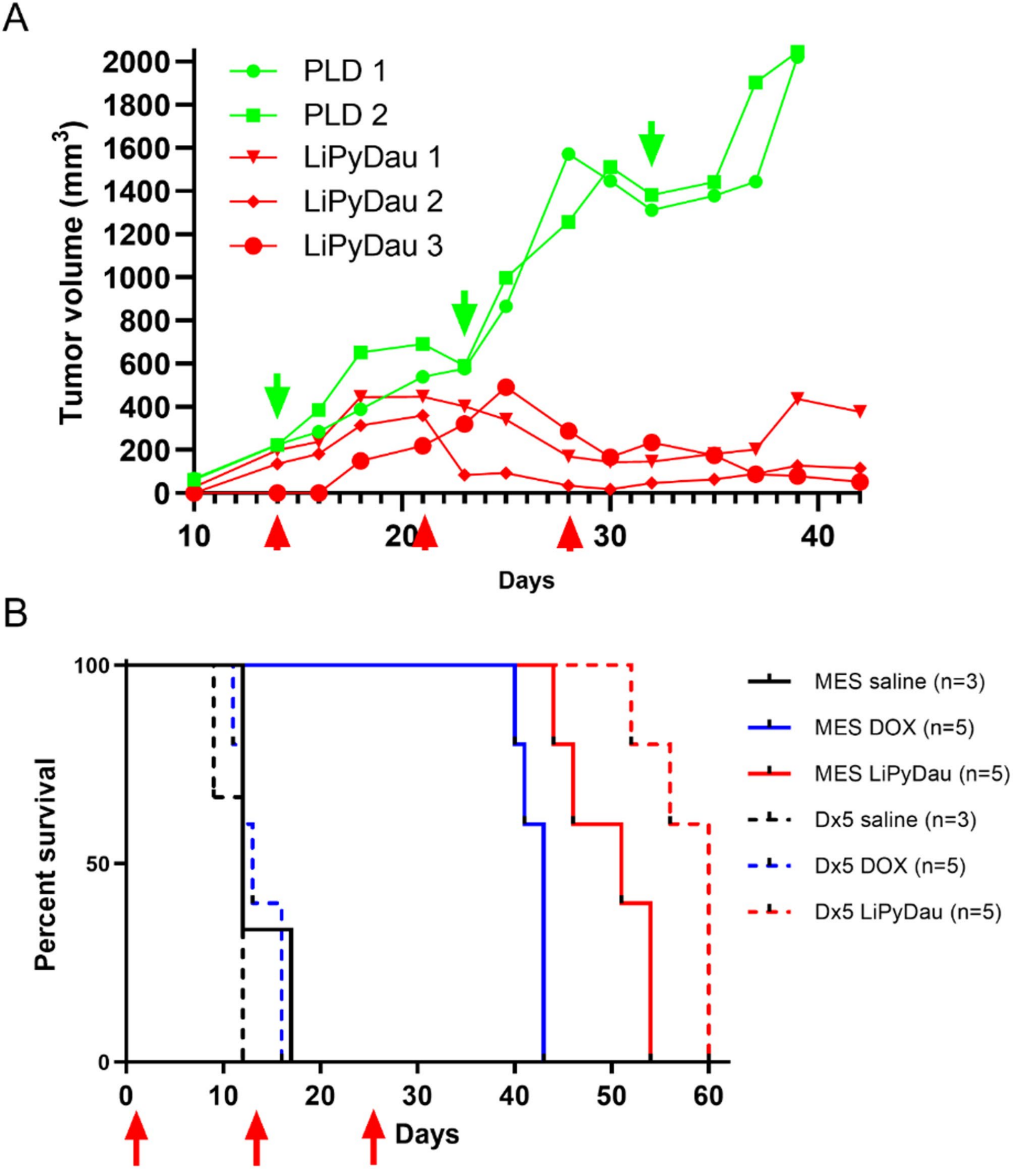
LiPyDau overcomes multidrug resistance in vivo. **A** Growth kinetics of PLD-resistant Brca1^−/−^;p53^−/−^ tumors engrafted into the mammary fat pad of wild-type female FVB mice. Mice were treated with 8 mg/kg PLD (green) or 1.5 mg/kg LiPyDau (distributed over 21 days (0.5 mg/kg/week), red). Treatments are indicated by arrows. **B** Overall survival of mice engrafted with human MES-SA (continuous lines) or its multidrug resistant derivative MES-SA/Dx5 cells (dashed lines), treated by saline (black), doxorubicin (5 mg/kg, blue) 1.5 mg/kg LiPyDau (distributed over 21 days (0.5 mg/kg/week), red). Treatments are indicated by arrows

Failure of eradication of Brca1^−/−^;p53^−/−^ tumors by DOX is due to P-gp mediated resistance [40]. In the same GEM model, resistance to pegylated liposomal DOX (PLD) also emerges, with significantly higher levels of P-gp in the refractory tumor cells [41, 42]. In vitro, PyDau remained highly toxic to MDR cells (Fig. 1B). In the next set of experiments, we assessed the potential of LiPyDau treatment in overcoming multidrug resistance. Tumor pieces harvested from PLD-resistant mice [41] were engrafted into the mammary fat pad of wild-type female FVB mice, and the mice were treated with either PLD or LiPyDau. As expected, PLD-resistant tumors failed to respond to PLD. Significantly, treatment with LiPyDau resulted in a pronounced antitumor effect (Fig. 6A). Finally, LiPyDau was tested against xenografts established from human MES-SA/Dx5 cells, which display extreme in vitro resistance to DOX as compared to the parental MES-SA cell line [43] (Fig. 1C). As shown in Fig. 6B, DOX treatment significantly prolongs the overall survival of mice engrafted with MES-SA cells, but it has no effect on Dx5 xenografts. In sharp contrast, treatment with LiPyDau was equally efficient in both xenografts, indicating that it can overcome multidrug resistance.

## Discussion

While anthracyclines are among the most effective anticancer drugs for cancer therapy, drug resistance and cardiotoxicity limit their clinical application. Since the first isolation of doxorubicin (DOX) and daunorubicin (DAU) from *Streptomyces peucetius* in the 1960s, several analogues have been reported, but only a few, such as idarubicin [44] and epirubicin [45] earned clinical approval.

In this study, we introduce a daunorubicin analog with extremely potent cytotoxic activity, building on the pioneering work of Nagy et al., who first developed 2-pyrrolinodoxorubicin for incorporation into targeted cytotoxic analogs of luteinizing hormone-releasing hormone for cancer therapy [13]. While PyDau exhibited in vitro toxicity in the nanomolar range, its extreme potency precluded any in vivo application. Liposomal encapsulation successfully expanded the therapeutic window, enabling safe administration of otherwise highly toxic doses, which translated into profound and long-lasting anticancer effects across multiple in vivo preclinical models.

When compared to other experimental anticancer agents, LiPyDau stands out due to its robust efficacy, delivering curative outcomes in several aggressive tumor models, including the genetically engineered Brca1^−/−^;p53^−/−^ breast cancer model (KBP1) as well as patient-derived xenografts of lung adenocarcinoma, which further underscores its translational potential. Furthermore, the formulation’s ability to overcome P-glycoprotein-mediated resistance, as observed also in in vivo experiments, highlights its promise as a next-generation therapeutic for combating refractory cancers.

BRCA-deficient tumors are known to exhibit increased sensitivity to DNA-damaging agents. Accordingly, mammary tumors in the KB1P mouse model for hereditary breast cancer show strong responsiveness to platinum drugs and PARP inhibitors [46, 47]. However, despite this heightened sensitivity, not all cancer cells are eradicated due to transient phenotypic changes linked to early developmental programs, which endow drug-tolerant persister (DTP) cells with stem-like properties, resilience and dormancy [48, 49]. This phenotypic plasticity, combined with dynamic changes in the tumor microenvironment, enables repeated cycles of adaptation, eventually leading to acquired drug resistance that renders the tumor refractory to treatment.

Notably, the phenotype of DTP cells includes transient resilience against a wide array of anticancer drugs [50]. Our results obtained with the KB1P model indicate that even combination therapy with fluorouracil, epirubicin, and cyclophosphamide fails to eradicate tumors. Yet, eliminating residual disease is considered crucial for improving long-term treatment outcomes. The activity pattern of PyDau across cells lacking individual DNA repair components supports a distinct mode of cytotoxicity. Unlike DAU and DOX, which primarily act as topoisomerase II inhibitors, PyDau is likely to exert its effects through the formation of an aminal adduct with the amino group of a guanine base as observed in the case of 2-pyrrolinodoxorubicin [30]. Indeed, our experimental results and molecular docking simulations (presented in Figs. 1E-I and S3-4) indicate that PyDau intercalates among the base pairs of the DNA to form covalent bonds with one of the two strands, while interacting non-covalently with the other strand. This interaction is similar to the structural characteristics of the daunorubicin-formaldehyde DNA adduct [51], which are also defined by covalent bonding to one strand of DNA, hydrogen bonding with the complementary strand, and hydrophobic interactions with both strands. However, in contrast to daunorubicin-formaldehyde DNA adduct, which is prone to hydrolysis, readily reverting to its constituent elements [52], the reactive component of PyDau, specifically the hemiaminal warhead, is covalently linked to the daunosamine, which enhances the stability of the resultant adduct. Together, these effects lead to catastrophic replication stress and double-strand DNA breaks, ultimately driving potent anticancer activity.

Earlier studies suggested that drug-tolerant cells could still be eliminated if sufficient damage is inflicted [53]. While lower doses of LiPyDau induced long-lasting responses, tumors invariably relapsed, often retaining sensitivity to treatment. However, when LiPyDau was administered at the maximum tolerated dose (MTD) or at lower doses distributed over three weeks, tumors were eradicated, leading to the cure of mice. These results suggest that in the KB1P model, persister cells can be effectively targeted by LiPyDau, as their ability to repair interstrand crosslinks is impaired due to the permanent loss of BRCA1 function [53].

The development of nanoformulations, such as PEGylated liposomal carriers, has revolutionized the field of cancer therapeutics by enhancing drug solubility, stability, and delivery to tumor sites while mitigating systemic toxicity. Functionalized liposomal and polymeric formulations of anthracyclines offer several benefits, including efficient drug loading and encapsulation, sustained and controlled release, the ability to overcome key therapeutic challenges like multidrug resistance (MDR) [41] and the blood-brain barrier (BBB) in brain tumors, as well as enhanced targeted drug delivery [54]. In vitro experiments assessing PyDau release under simulated tumor conditions and in plasma (Supplementary Figures S8–S9), together with in vivo studies of intratumoral accumulation and pharmacokinetics (Supplementary Figure S10), indicate that LiPyDau achieves more sustained tumor retention and slower release kinetics than the clinically used PLD formulation—factors that likely contribute to its pronounced antitumor efficacy. LiPyDau demonstrates several additional advantages over clinically approved liposomal anticancer formulations, such as Doxil^®^ (PEGylated liposomal DOX) and Myocet^®^ (non-PEGylated liposomal doxorubicin). While Doxil^®^ has shown clinical success in reducing cardiotoxicity and improving drug delivery in cancers like ovarian and breast cancer, its efficacy can be compromised by multidrug resistance mechanisms [41, 55]. Similarly, Myocet^®^ has been employed to mitigate the cardiotoxic effects of DOX, but its application remains limited to certain cancer types [56]. In contrast, LiPyDau offers a distinct advantage by effectively overcoming P-glycoprotein-mediated drug resistance, which is a major limitation of conventional liposomal anthracyclines. Furthermore, LiPyDau achieves curative outcomes in aggressive and multidrug-resistant tumor models, showcasing its potential as a superior alternative to existing liposomal drugs. These findings emphasize the transformative role of LiPyDau in advancing liposomal drug delivery for cancer therapy.

The superior therapeutic performance of LiPyDau can be attributed to the combined benefits of the distinct mechanism of action and the nanoformulation strategy. Encapsulation within PEGylated liposomes not only protected PyDau from premature degradation/metabolism but also facilitated its preferential accumulation in tumors (Figure S10) due to the enhanced permeability and retention (EPR) effect, and other changes in the tumor endothelium [57]. These characteristics position LiPyDau as a notable advancement over traditional anthracyclines and even other experimental nanoformulated chemotherapeutics.

From a clinical perspective, the use of LiPyDau could address several key challenges in cancer treatment, including overcoming drug resistance and reducing the risk of cardiotoxicity associated with conventional anthracyclines. The ability to safely administer high doses with minimal systemic toxicity opens the door to potential applications in treating high-risk cancer patients who currently lack effective therapeutic options. Moving forward, further clinical development and evaluation in human trials will be critical to establish the safety and efficacy of LiPyDau, setting the stage for its potential translation to clinical practice.

## Supplementary Information

Below is the link to the electronic supplementary material.

Supplementary Material.

## Data Availability

No datasets were generated or analysed during the current study.

## Materials availability

The newly developed reagent LiPyDau described in this work has been patented (Patent No. EP4236917, 2025) and is available from the corresponding author upon reasonable request, subject to completion of a material transfer agreement.
