# Supplementary material for: Safe delivery of a highly toxic anthracycline derivative through liposomal nanoformulation achieves complete cancer regression
Source: Mol Cancer. 2025 Oct 27;24:269. doi: 10.1186/s12943-025-02444-1 (PMC12557964; doi:10.1186/s12943-025-02444-1)
Supplement: Supplementary file 1 — Supplementary Material. [file 12943_2025_2444_MOESM1_ESM.pdf]

Safe delivery of a highly toxic anthracycline derivative through liposomal nanoformulation achieves complete cancer regression

András Füredi[1,2,3,4*], Szilárd Tóth[1,3*], Kristóf Hegedüs[5,6*], Pál T. Szabó[7], Anikó Gaál[8], Gergő Barta[8], Lívia N. Naszályi[8], Krisztina Kiss[6,9], Kata Bölcskei[10], Zoltán Szeltner[1], Eszter Bajtai[1,11], Balázs Gombos[1,11], Dániel Kiss[12], Mihály T. Cserepes[13,14], Attila Kiss[15], Peter Pokreisz[15], Lukas Kenner[16,17], Sandra Högler[17], Csaba Magyar[1], Jamie D. Cowles[18], Agnes Csiszar[18], József Tóvári[13], Dávid Szüts[1], Zsuzsanna Helyes[3,10,19,20], Zoltán Varga[8,21], Gábor Mező[6,9], Gergely Szakács[1,3,18,†]

[1]Institute of Molecular Life Sciences, HUN-REN Research Centre for Natural Sciences, H-1117 Budapest, Hungary

[2]Institute of Technical Physics and Materials Science, HUN-REN Centre of Energy Research, H-1121 Budapest, Hungary

[3]National Laboratory for Drug Research and Development, H-1117 Budapest, Hungary

[4]Physiological Controls Research Center, University Research and Innovation Center, Óbuda University, H-1034 Budapest, Hungary

[5]Organocatalysis Research Group, Institute of Organic Chemistry, HUN-REN Research Centre for Natural Sciences, H-1117 Budapest, Hungary

[6]Department of Organic Chemistry, Institute of Chemistry, Faculty of Science, ELTE Eötvös Loránd University, H-1117 Budapest, Hungary

[7]Centre for Structural Science, HUN-REN Research Centre for Natural Sciences, H-1117 Budapest, Hungary

[8]Institute of Materials and Environmental Chemistry, HUN-REN Research Centre for Natural Sciences, H-1117 Budapest, Hungary

[9]HUN-REN-ELTE Research Group of Peptide Chemistry, H-1117 Budapest, Hungary

[10]Department of Pharmacology and Pharmacotherapy, Medical School, University of Pécs, H-7624, Pécs, Hungary

[11]Semmelweis University Doctoral School, H-1085 Budapest, Hungary

[12]John von Neumann Faculty of Informatics, Óbuda University, Budapest, Hungary

[13]Department of Experimental Pharmacology and the National Tumor Biology Laboratory, National Institute of Oncology, Budapest, Hungary

[14]KINETO Lab Ltd, H-1037, Budapest, Hungary

[15]Center for Biomedical Research and Translational Surgery, Medical University of Vienna, Vienna, Austria

[16]Clinical Institute of Pathology, Department for Experimental and Laboratory Animal Pathology, Medical University of Vienna, Vienna, Austria

[17]Unit of Laboratory Animal Pathology, University of Veterinary Medicine Vienna, Vienna, Austria

[18]Center for Cancer Research, Medical University of Vienna, Vienna, Austria

[19]HUN-REN-PTE Chronic Pain Research Group, University of Pécs, H-7624, Pécs, Hungary

[20]PharmInVivo Ltd, H-7629, Pécs, Hungary

[21]Department of Physical Chemistry and Materials Science, Budapest University of Technology and Economics, H-1111 Budapest, Hungary

*These authors contributed equally to this work.
†Corresponding author

Table of contents

Supplementary Figure S1. Synthesis and characterization of pyrrolino-daunorubicin (PyDau)
Supplementary Figure S2. Preparation and characterization of α-methyl-2-pyrrolino-daunorubicin (MePyDau)
Supplementary Table S1. IC_{50} values of daunomycin (DAU), doxorubicin (DOX) and 2-pyrrolino-daunomycin (PyDau) against human cell lines
Supplementary Table S2. Relative IC_{50} values and standard deviation (+SD/-SD) of chemotherapeutic drugs against DNA repair KO variants of DT40 cell line
Supplementary Figure S3. Molecular docking and simulation studies confirm the strong binding affinity of PyDau to DNA.
Supplementary Figure S4. PyDau cross-links the two strands of dsDNA in a denaturation resistant way
Supplementary Table S3. Mortality rates and survival of free PyDau treated FVB mice
Supplementary Table S4. Mortality rates and survival of LiPyDau treated FVB mice
Supplementary Figure S5. Body weight changes related to LiPyDau treatment in different tumor models and mouse strains.
Supplementary Figure S6. Chronic toxicity of LiPyDau treatment
Supplementary Figure S7. Acute toxicity of LiPyDau treatment
Supplementary Figure S8. In vitro drug release profile of LiPyDau under tumor-relevant conditions.
Supplementary Figure S9. Plasma stability of LiPyDau
Supplementary Figure S10. Pharmacokinetic profile of LiPyDau in plasma and tumor tissue.

Supplementary Figure S1. Synthesis and characterization of pyrrolino-daunorubicin (PyDau)

All reactions were carried out using oven-dried glassware and anhydrous solvents, under an atmosphere of nitrogen unless noted otherwise. HRMS spectra were obtained using Thermo Fisher Scientific Q-Exactive Focus hybrid quadrupole-orbitrap mass spectrometer equipped with heated electrospray ionization source Under the applied conditions, the compounds form protonated molecules, $[M + H]^+$. ^{1}H- and ^{13}C-NMR spectra were recorded using a Varian 500 MHz INOVA spectrometer. Chemical shifts are referenced to the residual solvent signals ($CDCl_3$: δ = 7.26 ppm for ^{1}H, δ = 77.0 ppm for ^{13}C). Data are reported as follows: chemical shifts (ppm), multiplicity (s = singlet, d = doublet, t = triplet, q = quartet, br = broad, m = multiplet), and coupling constants (Hz). All spectra were recorded with the standard spectrometer pulse sequences and settings, ^{1}H decoupling was used for ^{13}C-NMR measurements.

Synthesis of PyDau

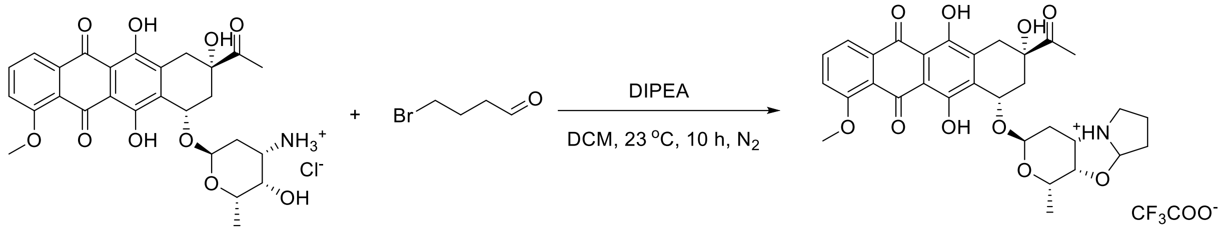

To a solution of *N,N*-diisopropylethylamine (1.00 g, 7.74 mmol, 4.35 equiv) in dichloromethane (100 mL) was added daunorubicin hydrochloride (1.00 g, 1.77 mmol, 1 equiv) at 23 °C under nitrogen atmosphere. The suspension was stirred vigorously until the hydrochloride salt was completely dissolved. The resulting deep purple solution was stirred for 15 minutes at the same temperature and 4-bromobutyraldehyde (0.54 g, 3.5 mmol, 2.0 equiv) was added in one portion. The reaction mixture was stirred until high-performance liquid chromatography analysis indicated full conversion of daunorubicin (6–10 h). The reaction mixture was concentrated under reduced pressure (20–25 mL) and the resulting deep red oil was diluted with diethyl ether (100 mL). The resulting suspension was stirred for 10 minutes at the same temperature and decanted. The procedure of dilution, stirring and decantation was repeated two more times. The resulting red slurry was dissolved in methanol (25 mL), and trifluoroacetic acid (1 mL) was added dropwise at 5 °C. The solution was filtered and the filtrate was concentrated. The residue was purified by crystallization from diethyl ether (50 mL). The amorphous solid was washed with diethyl ether (2 × 50 mL) and dried under reduced pressure to yield trifluoroacetate (1.00 g, HPLC purity = 80%, 1.15 mmol, 65.2%) as a red solid.

The raw product was further purified via HPLC to yield the trifluoroacetate salt.

A small sample from the free base was purified by flash column chromatography on silica gel (0% methanol in dichloromethane grading to 10% methanol in dichloromethane) for characterization.

The hydrochloride salt was prepared from the 0.1 M dichloromethane solution of the free base by adding equimolar 2M ethereal hydrogen chloride solution, followed by evaporation. The raw salt was crystallized from ethyl acetate, followed by filtration. The red crystalline solid was washed with ethyl acetate and dried under reduced pressure to yield hydrochloride salt.

Physical State: deep red solid

^{1}H-NMR (500 MHz, $CDCl_3$) δ 13.77 (s, 2H), 13.07 (s, 2H), 7.89 (dt, *J* = 7.6, 1.3 Hz, 2H), 7.68 (td, *J* = 8.3, 1.4 Hz, 2H), 7.31 (dd, *J* = 8.6, 1.2 Hz, 2H), 5.45 (t, *J* = 4.7 Hz, 2H), 5.27 (d, *J* = 1.4 Hz, 1H), 5.21 (dd, *J* = 4.0, 2.1 Hz, 2H), 5.12 (dd, *J* = 5.3, 1.8 Hz, 2H), 4.75 (s, 1H), 4.06 (qd, *J* = 6.4, 1.8 Hz, 2H), 4.02 (s, 3H), 3.85 (dd, *J* = 5.9, 1.9 Hz, 2H), 3.32 – 3.25 (m, 2H), 3.08 – 3.07 (m, 1H), 3.07 (d, *J* = 18.8 Hz, 2H), 2.78 (d, *J* = 18.8 Hz, 2H), 2.58 (ddd, *J* = 11.1, 8.7, 6.8 Hz, 2H), 2.42 (t, *J* = 2.0 Hz, 1H), 2.40 (s, 0H), 2.38 (s, 3H), 2.05 – 2.04 (m, 1H), 2.03 (s, 0H), 1.86 – 1.85 (m, 1H), 1.80 – 1.79 (m, 1H), 1.71 – 1.70 (m, 1H), 1.70 – 1.69 (m, 1H), 1.34 (d, *J* = 6.6 Hz, 3H).

^{13}C NMR (126 MHz, $CDCl_3$) δ 212.0, 186.6, 186.4, 161.0, 156.4, 155.7, 135.6, 135.4, 134.5, 134.3, 120.8, 119.7, 118.4, 111.2, 111.1, 100.8, 97.4, 77.4, 77.1, 76.9, 76.9, 74.2, 69.4, 65.6, 58.9, 56.6, 54.4, 34.9, 33.3, 32.2, 31.3, 24.8, 24.3, 17.4.

HRMS (ESI): calcd. for $C_{31}H_{34}NO_{10}^{+}$ $[M+H]^{+}$ 580.2177, found 580.2166.

^{1}H-NMR (500 MHz, $CDCl_3$) spectra of **PyDau**

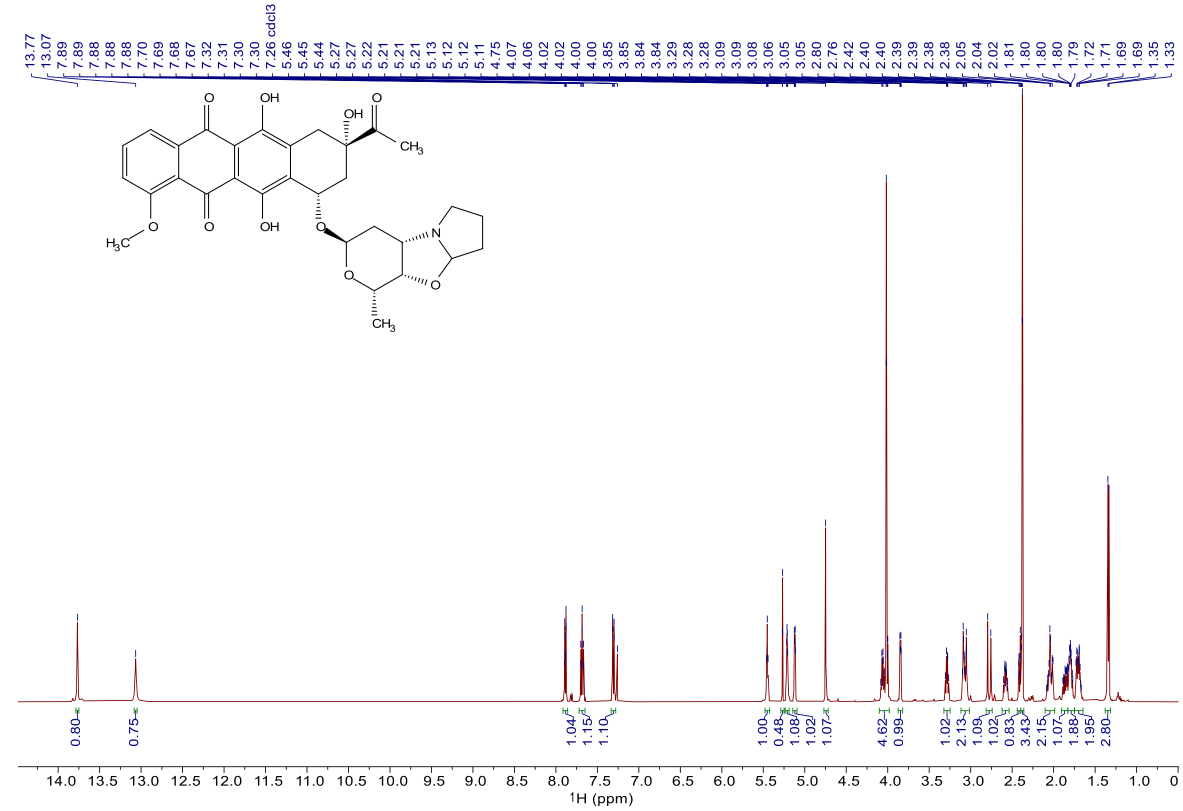

^{13}C-NMR (126 MHz, $CDCl_3$) spectra of **PyDau**

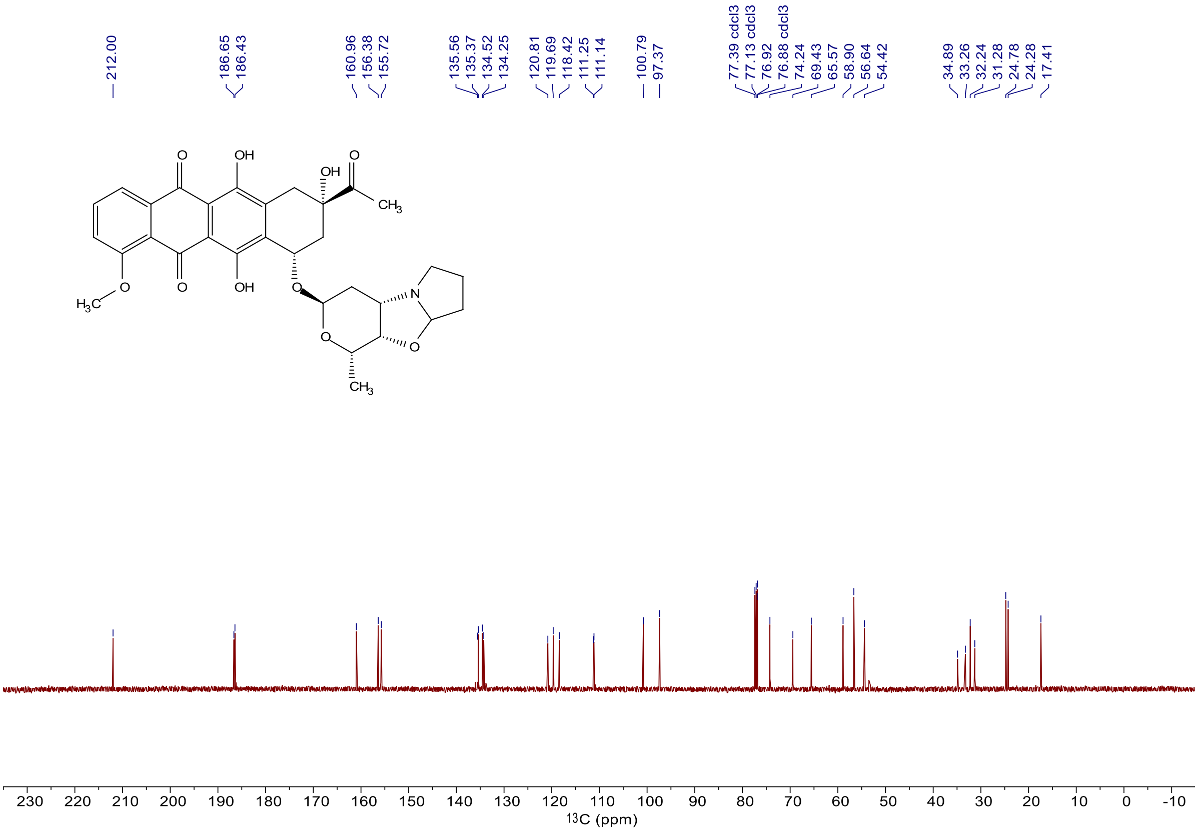

Supplementary Figure S2. Preparation and characterization of α-methyl-2-pyrrolino-daunorubicin (MePyDau):

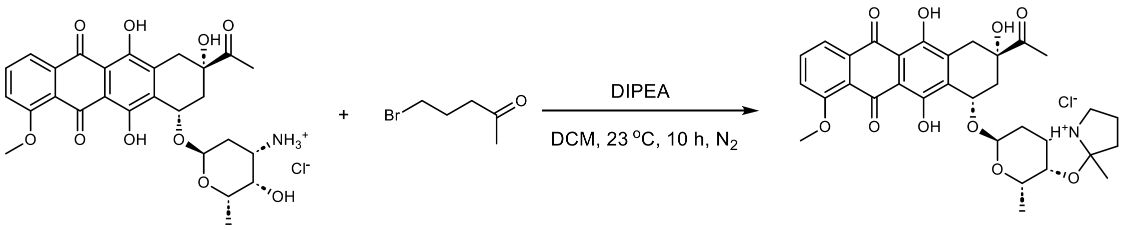

To a solution of *N,N*-diisopropylethylamine (1.00 g, 7.74 mmol, 4.35 equiv) in dichloromethane (100 mL) was added daunorubicin hydrochloride (1.00 g, 1.77 mmol, 1 equiv) at 23 °C under nitrogen atmosphere. The suspension was stirred vigorously until the hydrochloride salt was completely dissolved. The resulting deep purple solution was stirred for 15 minutes at the same temperature and 5-bromopentan-2-one (0.58 g, 3.5 mmol, 2.0 equiv) was added in one portion. The reaction mixture was stirred until high-performance liquid chromatography analysis indicated full conversion of daunorubicin (24–48 h). The reaction mixture was concentrated under reduced pressure (20–25 mL) and the resulting deep red oil was diluted with diethyl ether (100 mL). The resulting suspension was stirred for 10 minutes at the same temperature and decanted. The procedure of dilution, stirring and decantation was repeated two more times. The raw product was dissolved in dichloromethane (100 ml), evaporated on Celite and purified by flash column chromatography on silica gel (0% methanol in dichloromethane grading to 10% methanol in dichloromethane) to yield MePyDau as a red solid (505 mg, 0.85 mmol, 48%).

The hydrochloride salt was prepared from the 0.1 M dichloromethane solution of the free base by adding equimolar 2M ethereal hydrogen chloride solution, followed by evaporation. The raw salt was crystallized from ethyl acetate, followed by filtration. The red crystalline solid was washed with ethyl acetate and dried under reduced pressure to yield hydrochloride salt.

Physical State: deep red solid

^{1}H NMR (500 MHz, $CDCl_3$) δ 13.86 (s, 1H), 13.16 (s, 1H), 7.96 (dd, J = 7.7, 1.1 Hz, 1H), 7.74 (t, J = 8.1 Hz, 1H), 7.36 (dd, J = 8.6, 1.0 Hz, 1H), 5.49 (t, J = 3.4 Hz, 1H), 5.27 (dd, J = 4.0, 2.2 Hz, 1H), 4.76 (s, 1H), 4.22 (qd, J = 6.6, 2.3 Hz, 1H), 4.06 (s, 3H), 3.78 (dd, J = 5.0, 2.3 Hz, 1H), 3.18 – 3.18 (m, 1H), 3.16 – 3.15 (m, 1H), 2.99 (td, J = 8.3, 4.9 Hz, 1H), 2.87 (d, J = 18.8 Hz, 1H), 2.62 (td, J = 9.8, 5.5 Hz, 1H), 2.41 (s, 3H), 2.44 – 2.36 (m, 1H), 2.08 (dd, J = 14.8, 4.0 Hz, 1H), 1.91 – 1.83 (m, 1H), 1.82 – 1.79 (m, 2H), 1.78 – 1.76 (m, 1H), 1.76 – 1.74 (m, 1H), 1.69 – 1.60 (m, 1H), 1.49 (s, 3H), 1.39 (d, J = 6.7 Hz, 3H).

^{13}C NMR (126 MHz, $CDCl_3$) δ 211.7, 186.7, 186.5, 161.0, 156.4, 155.7, 135.6, 135.4, 134.4, 134.3, 120.9, 119.7, 118.4, 111.3, 111.2, 104.4, 101.0, 77.3, 77.0, 76.9, 76.8, 73.2, 69.5, 64.8, 58.2, 56.6, 55.2, 38.7, 34.8, 33.4, 31.8, 29.3, 24.7, 24.1, 17.4.
HRMS (ESI): calcd. for $C_{32}H_{36}NO_{10}^+$ $[M+H]^+$ 594.2334, found 594.2322.

¹H-NMR (500 MHz, $CDCl_3$) spectra of **MePyDau**

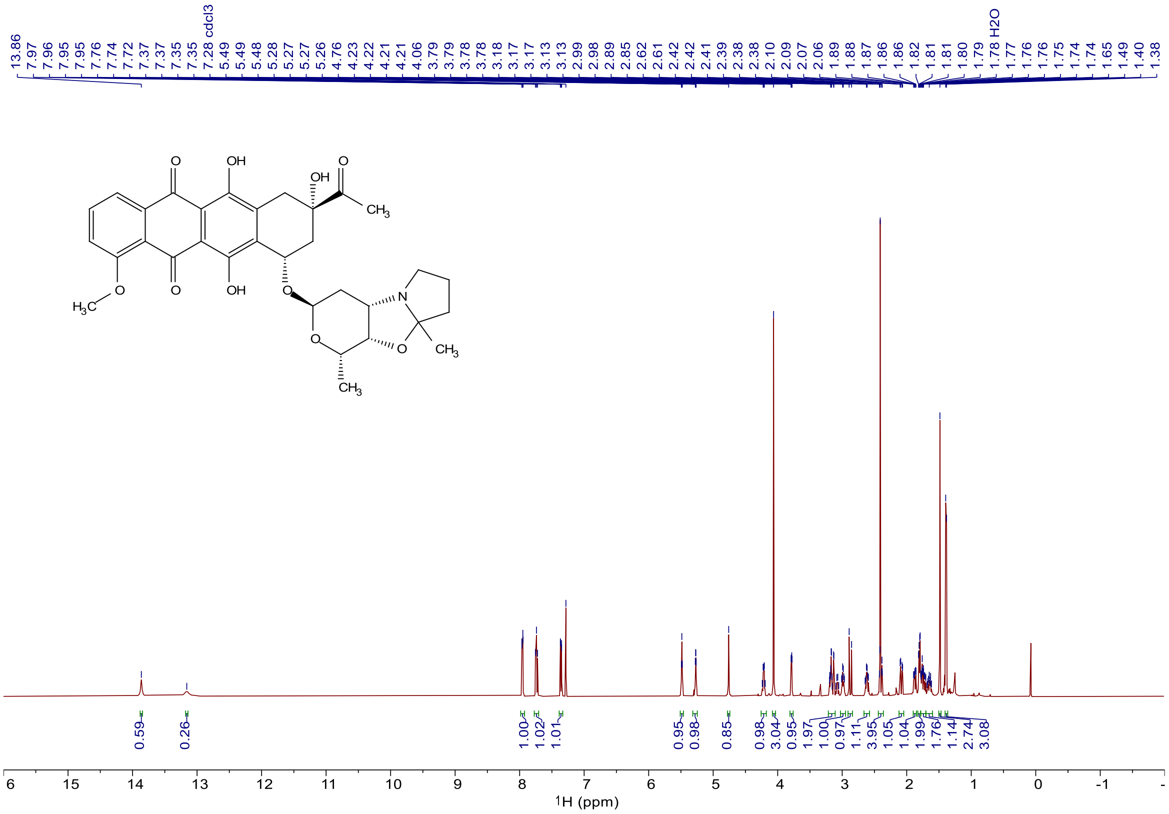

¹³C-NMR (126 MHz, $CDCl_3$) spectra of **MePyDau**

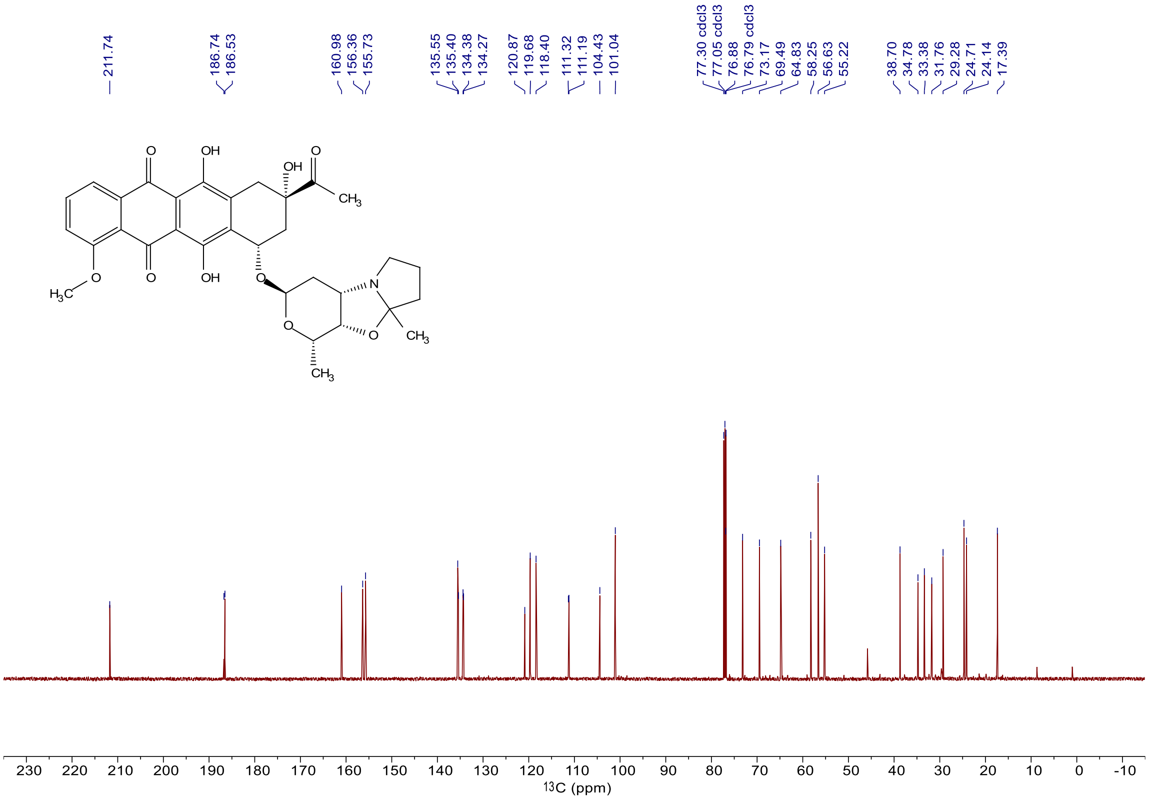

Supplementary Table S1. IC_{50} values of daunomycin (DAU), doxorubicin (DOX) and 2-pyrrolino-daunomycin (PyDau) against human cell lines expressed in nM. Cytotoxicity of DAU and DOX were taken from the NCI-60 database and from the literature[1,2] or measured with PrestoBlue viability assay for DLD-1 and 143B. Cytotoxicity of PyDau was measured with PrestoBlue reagent and presented with the respective standard deviations (+SD/-SD). Cell lines are grouped based on their tissue of origin.

	DAU	DOX	PyDau	+SD/-SD
MALME-3M	49.7	51.9	1.84	0.66/0.48
SK-MEL-28	132.1	242.1	6.87	0.82/0.74
SK-MEL-2	157.8	236.6	3.55	1.43/1.02
HT29	50.7	171.4	3.53	1.59/1.10
DLD-1	59.6	40.4	2.57	1.10/0.77
HCT-15	650.1	1202.3	2.42	0.90/0.66
COLO 205	52.5	158.1	2.38	0.51/0.42
HCC-2998	99.3	180.7	8.19	3.40/2.40
HCT-116	28.6	66.1	3	2.05/1.22
KM12	95.9	223.4	1.42	0.38/0.30
SW-620	35.5	70.1	2.78	0.60/0.49
EKVX	359.7	484.2	3.67	0.90/0.72
NCI-H522	36.1	54.7	0.94	0.46/0.31
NCI-H1792	n.d.	n.d.	14.28	3.31/2.26
NCI-H322M	288.4	452.9	7.85	3.84/3.22
LCLC	n.d.	n.d.	12.65	2.52/1.80
HOP-62	53	60.4	4.16	2.34/1.10
NCI-H226	46.5	52.4	4.44	1.29/1.00
PC-3	107.6	246	2.54	0.89/0.66
DU-145	76.7	100.2	1.31	0.93/0.64

	DAU	DOX	PyDau	+SD/-SD
OVCAR-5	155.2	587.5	1.93	1.22/0.75
OVCAR-3	124.5	335	1.22	0.70/0.45
IGROV-1	31.3	103.5	1.27	0.12/0.11
OVCAR-8	41.6	130.3	1.19	0.81/0.48
NCI/ADR-RES	1230.3	13552	1.38	0.64/0.44
UO-31	174.6	553.4	1.43	0.64/0.44
A498	137.1	85.5	13.3	4.15/3.16
CAKI-1	228.6	189.7	1.4	0.57/0.31
RXF 393	92.5	159.6	4.66	2.02/1.41
MDA-MB-231	200.9	382.8	1.09	0.79/0.46
MCF7	19.5	15.6	5.73	1.98/1.66
T-47D	40.8	83	4.25	2.41/1.74
MDA-MB-435	136.8	257.6	1.86	0.95/0.47
HS 578T	119.7	188.4	4.52	1.73/0.98
BT-549	153.1	212.3	2.89	1.74/1.09
PANC-1	260	460	1.77	1.22/0.72
Bx-PC-3	n.d.	n.d.	4.68	2.16/1.12
143B	n.d.	107.2	0.41	0.13/0.10
HL-60	31.2	62.4	0.9	0.39/0.27
CCRF-CEM	22.8	37.2	0.23	0.35/0.14

Supplementary Table S2. Relative IC_{50} values and standard deviation (+SD/-SD) of chemotherapeutic drugs against DNA repair KO variants of DT40 cell line, normalized to the IC_{50} values of wild type DT40 (WT). Cytotoxicity was measured by PrestoBlue viability assay. Subset of the data was already published earlier[3].

	**WT**	**BRCA1**	**BRCA2**	**PALB2**	**RAD51C**	**XRCC2**	**XRCC3**	**RAD52**	**RAD54**	**ATM**	**CHK2**	**REV1**	**KU70**
Olaparib	1.00	0.01	0.02	0.06	0.13	0.46	0.36	0.68	0.12	0.53	3.74	0.57	1.45
	0.07/0.06	*0.00/0.00*	*0.01/0.01*	*0.02/0.02*	*0.03/0.02*	*0.08/0.07*	*0.09/0.07*	*0.13/0.11*	*0.03/0.02*	*0.06/0.05*	*0.82/0.67*	*0.11/0.09*	*0.26/0.22*
Cisplatin	1.00	0.44	0.17	0.51	0.39	0.46	0.19	0.17	0.47	1.29	4.17	0.01	1.38
	0.11/0.10	*0.07/0.06*	*0.03/0.03*	*0.10/0.08*	*0.10/0.08*	*0.05/0.04*	*0.06/0.05*	*0.03/0.02*	*0.03/0.03*	*0.28/0.23*	*0.67/0.58*	*0.00/0.00*	*0.16/0.15*
Daunomycin	1.00	0.74	0.86	1.11	1.17	1.44	1.23	0.91	0.61	1.77	1.27	0.68	0.19
	0.11/0.10	*0.13/0.11*	*0.27/0.20*	*0.08/0.07*	*0.19/0.17*	*0.33/0.27*	*0.20/0.17*	*0.19/0.16*	*0.16/0.13*	*0.34/0.28*	*0.13/0.12*	*0.08/0.07*	*0.04/0.03*
PyDau	1.00	0.17	0.09	0.18	0.23	0.75	0.38	0.89	0.16	1.03	2.41	1.00	0.88
	0.14/0.13	*0.03/0.02*	*0.05/0.03*	*0.13/0.07*	*0.11/0.07*	*0.26/0.19*	*0.20/0.13*	*0.14/0.12*	*0.08/0.05*	*0.26/0.21*	*0.44/0.37*	*0.31/0.24*	*0.25/0.19*
SN-38	1.00	0.10	0.39	0.46	0.62	1.21	1.04	0.70	0.46	0.58	4.03	0.44	3.48
	0.06/0.06	*0.01/0.01*	*0.06/0.05*	*0.07/0.06*	*0.09/0.08*	*0.17/0.15*	*0.08/0.08*	*0.09/0.08*	*0.07/0.06*	*0.05/0.05*	*1.23/0.94*	*0.08/0.07*	*0.82/0.66*
Etoposide	1.00	0.45	0.38	0.59	0.96	1.60	1.42	1.37	0.47	1.62	6.15	0.94	0.08
	0.08/0.07	*0.05/0.05*	*0.01/0.01*	*0.08/0.07*	*0.08/0.08*	*0.06/0.06*	*0.15/0.14*	*0.04/0.04*	*0.89/0.78*	*0.03/0.03*	*3.06/2.64*	*0.08/0.08*	*0.18/0.15*
Hydroxyurea	1.00	1.21	1.23	1.21	1.11	1.04	0.84	1.58	0.83	1.32	1.34	0.97	0.83
	0.07/0.07	*0.08/0.07*	*0.10/0.09*	*0.11/0.10*	*0.09/0.08*	*0.14/0.12*	*0.25/0.19*	*0.19/0.17*	*0.05/0.05*	*0.22/0.19*	*0.12/0.11*	*0.12/0.11*	*0.10/0.09*
Gemcitabine	1.00	0.80	0.64	0.97	0.60	0.99	0.77	1.12	0.97	1.19	1.37	0.87	1.04
	0.09/0.08	*0.09/0.08*	*0.07/0.06*	*0.05/0.05*	*0.09/0.08*	*0.07/0.06*	*0.05/0.05*	*0.09/0.08*	*0.05/0.05*	*0.01/0.01*	*0.12/0.11*	*0.11/0.09*	*0.13/0.11*
Paclitaxel	1.00	0.95	0.99	0.97	1.22	1.49	0.82	0.88	0.83	1.54	1.68	1.59	1.44
	0.18/0.15	*0.28/0.22*	*0.25/0.20*	*0.21/0.17*	*0.22/0.19*	*0.38/0.30*	*0.21/0.17*	*0.12/0.11*	*0.23/0.18*	*0.47/0.36*	*0.52/0.40*	*0.19/0.17*	*0.18/0.16*
Temozolomide	1.00	0.44	0.53	0.57	0.82	1.02	0.82	0.54	0.37	0.78	1.30	0.04	*n.d.*
	0.10/0.09	*0.05/0.04*	*0.10/0.09*	*0.06/0.06*	*0.03/0.03*	*0.09/0.09*	*0.09/0.08*	*0.03/0.03*	*0.04/0.04*	*0.06/0.05*	*0.14/0.12*	*0.00/0.00*	*n.d.*
Talazoparib	1.00	0.00	0.01	0.01	0.03	0.24	0.12	0.21	0.03	0.13	16.51	0.53	3.16
	0.20/0.16	*0.00/0.00*	*0.00/0.00*	*0.00/0.00*	*0.00/0.00*	*0.09/0.06*	*0.04/0.03*	*0.03/0.03*	*0.00/0.00*	*0.02/0.02*	*6.18/4.49*	*0.06/0.06*	*0.39/0.34*
Carboplatin	1.00	0.33	0.16	0.42	0.24	0.45	0.18	0.13	0.44	1.41	3.28	0.02	*n.d.*
	0.06/0.06	*0.02/0.02*	*0.01/0.01*	*0.00/0.00*	*0.02/0.02*	*0.03/0.03*	*0.01/0.01*	*0.03/0.02*	*0.06/0.05*	*0.06/0.05*	*0.40/0.35*	*0.00/0.00*	*n.d.*
Oxaliplatin	1.00	0.43	0.95	1.08	0.76	0.47	0.41	0.22	0.45	0.71	0.89	0.03	*n.d.*
	0.10/0.09	*0.06/0.05*	*0.13/0.12*	*0.30/0.23*	*0.05/0.05*	*0.04/0.04*	*0.05/0.04*	*0.04/0.03*	*0.02/0.02*	*0.07/0.07*	*0.11/0.10*	*0.00/0.00*	*n.d.*
Doxorubicin	1.00	0.67	0.64	1.08	1.54	1.43	1.48	0.81	0.62	1.37	2.95	0.19	0.08
	0.10/0.09	*0.02/0.02*	*0.04/0.04*	*0.19/0.16*	*0.18/0.16*	*0.23/0.20*	*0.15/0.14*	*0.17/0.14*	*0.07/0.06*	*0.03/0.03*	*0.86/0.67*	*0.17/0.09*	*0.00/0.00*

Supplementary Figure S3. Molecular docking and simulation studies confirm the strong binding affinity of PyDau to DNA.

In Schrödinger's CovDock, docking scores represent the predicted binding affinity of a molecule to a target, specifically taking into account the formation of a covalent bond. These scores are crucial for identifying potential drug candidates that can form a stable, covalent bond with a target protein, a common mechanism for some drugs. The lower (more negative) the score, the stronger the predicted binding affinity, indicating a more favorable interaction. In order to further validate the predicted binding modes of PyDau to DNA (see Figure 1I), we performed Binding Pose Metadynamics (BPMD) simulations[4]. The simulation using the top-ranked docking pose yielded a low RMSD value (1.2 Å), indicating that the predicted complex is stable and energetically favorable (Fig. S3A). In contrast, the second-ranked pose, which involved only a single hydrogen bond, exhibited a much higher RMSD throughout the BPMD trajectory (Fig. S3B). The jagged nature of the latter RMSD curve suggests that the ligand attempted to dissociate from the binding site during the simulation, but was retained by the covalent bond. These results support the viability and stability of the top-ranked pose shown in Figure 1I as the most plausible binding conformation of PyDau to DNA.

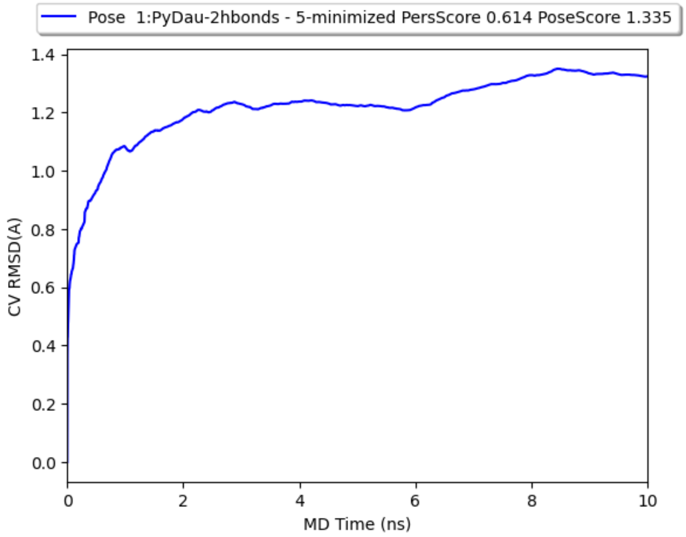

Figure S3A. Result of the BPMD simulation for the top-ranked PyDau–DNA binding pose. The low and stable RMSD values indicate a stable complex and support the reliability of the predicted binding conformation.

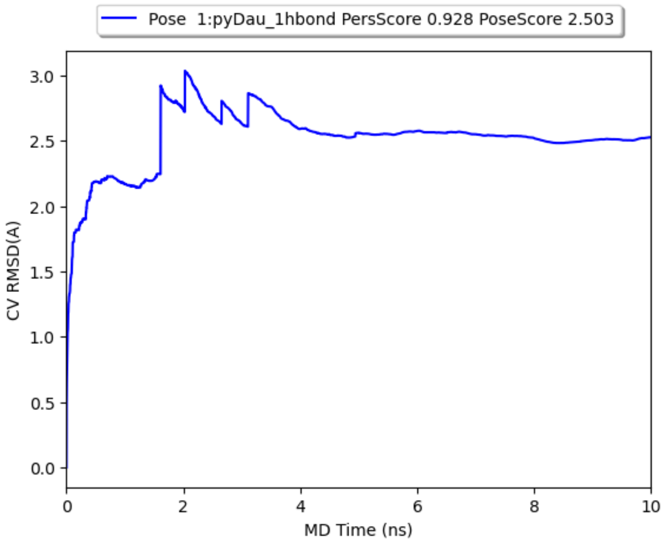

Figure S3B. Result of the BPMD simulation for the second-ranked PyDau–DNA binding pose.

To further validate the reliability of our CovDock-based modeling approach, we performed a control docking study using daunorubicin with a formaldehyde linker, re-docked into the DNA structure from the 1D33 PDB entry. In this simulation, the PyDau structure was treated as fully flexible, allowing exploration of various conformations. The best-ranking pose yielded a docking score of –9.02 kcal/mol and closely matched the experimentally determined structure, with an RMSD of 0.64 Å (Fig. S3C). These results demonstrate that CovDock accurately models the known daunomycin-DNA interaction, thereby supporting the credibility of our modeled PyDau-DNA complex.

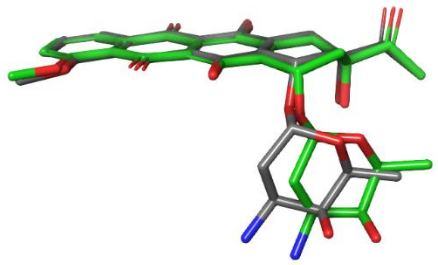

Figure S3C. Overlay of the crystal structure and the re-docked pose of daunorubicin. The re-docked complex closely matches the experimental structure (PDB ID: 1D33), with an RMSD of 0.64 Å, supporting the reliability of the docking method.

The BPMD simulation of the daunorubicin–DNA complex resulted in a higher RMSD compared to the top-ranked PyDau–DNA pose, indicating lower structural stability. This supports the

conclusion that PyDau forms a more stable DNA complex, which may contribute to its superior cytotoxic activity (Fig. S3D).

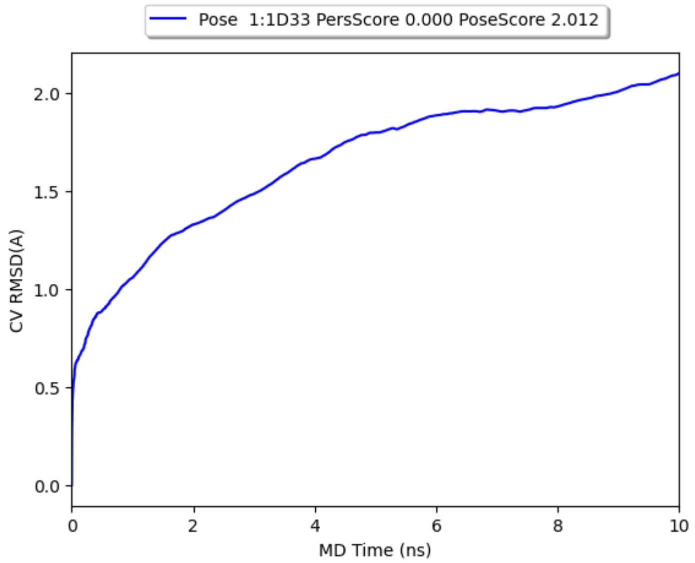

Figure S3D. Result of the BPMD simulation of the daunorubicin–DNA complex from the 1D33 PDB entry. The higher RMSD values observed during the simulation indicate reduced stability compared to the top-ranked PyDau–DNA complex.

Taken together, our model suggests that PyDau forms a covalent bond with one DNA strand and hydrogen bonds with the complementary strand, providing a plausible explanation for both the experimental results shown in Fig. 1 and the compound's potent antitumor activity.

Supplementary Figure S4. PyDau cross-links the two strands of dsDNA in a denaturation resistant way.

To assess the binding of PyDau to DNA and its effect on strand separation, we incubated PyDau and DAU with double-stranded DNA (dsDNA) constructs of various lengths and analyzed the outcomes using gel electrophoresis under both native and denaturing conditions. Specifically, comparative studies of daunorubicin (DAU), doxorubicin (DOX), and PyDau binding under denaturing conditions were performed using urea and alkaline gel electrophoresis with dsDNA constructs of different lengths (Fig. S4A-D). Additionally, we examined the reversibility of the denaturation-resistant interstrand cross-link formed by PyDau using a 318 bp dsDNA construct (Fig. S4C). Finally, the ability of DAU and PyDau to induce DNA cross-links under native conditions was evaluated with a 34 bp fluorescently labeled DNA construct via native polyacrylamide gel electrophoresis (Fig. S4E).

The results indicate significant differences in the binding potential of DAU (in some cases DOX) and PyDau:

(1) PyDau binds more strongly to the short 34 bp dsDNA construct under native conditions (Fig. S4 E) than under denaturing conditions (Fig. S4 B);

(2) only PyDau generates a shifted dsDNA band at both conditions (Fig. S4 B and E); and

(3) PyDau quenches the fluorescent GelRed dye at significantly lower concentrations (or reagent excess) than DAU, indicating its stronger binding to the DNA construct under native conditions (Fig. S4E). The complete loss of GelRed fluorescence further confirms that PyDau binding to DNA involves intercalation.

Resistance of cross-linked material to denaturants supports the possibility of covalent bond formation or a very strong, non-covalent cooperative binding mode of PyDau, potentially without formation of an aminal covalent bond. In both scenarios, the DNA–PyDau complex is expected to dissociate slowly. Indeed, we observed slow dissociation of the complex -as indicated by the gradual disappearance of the shifted DNA band - in the case of the 318 bp DNA following removal of excess PyDau and extensive dialysis (Supplementary figure 4C, lanes 10 and 12).

It is important to note that in conditions where excess reagent was removed after 5 hours of PyDau/DNA co-incubation via phenol-chloroform purification (see experiments shown in Fig. S4C and D), the lower mobility-shifted band persisted in the case of longer double-stranded DNAs (318 and 2880 bp), and not with the 34 bp constructs. The persistence of the lower mobility-shifted band is indicative of denaturation-resistant crosslink formation. Notably, this crosslinked product formed more readily with longer dsDNA and at lower PyDau excess as

DNA length increased (Fig. S4C and D). These findings suggest that only a few inseparable bonds hold the two DNA strands together, and such bonds are more likely to form at lower reagent concentrations in longer DNA molecules. As shown in Fig. S4D, a significant amount of this product was already detectable at a reagent/DNA base pair ratio as low as 0.006. The process appeared to reach completion at a tenfold higher excess (0.06 reagent/DNA bp), as illustrated in the figure (lane 5). When we tested a shorter, 318 bp dsDNA fragment, the fully developed lower mobility band indicative of interstrand cross-linking was only observed at nearly tenfold higher PyDau concentrations, corresponding to a reagent excess of 0.4 (Supplementary Figure S4C, lane 9). Extensive dialysis of the PyDau-saturated 318 bp DNA resulted in an almost complete disappearance of the low-mobility band, suggesting that the cross-linking is at least partially reversible under these conditions.

Based on the above, we suggest a model, in which PyDau intercalates more strongly and perhaps more intimately among the base pairs of the B-DNA and likely can form covalent bonds with one of the two strands, while interacting non-covalently with the other strand. This interaction is similar to the structural characteristics of the daunorubicin-formaldehyde DNA adduct[5], which are also defined by covalent bonding to one strand of DNA, hydrogen bonding with the complementary strand, and hydrophobic interactions with both strands. However, in contrast to daunorubicin-formaldehyde DNA adduct, which is prone to hydrolysis, readily reverting to its constituent elements[6], the reactive component of PyDau, specifically the hemiaminal warhead, is covalently linked to the daunosamine, which enhances the stability of the resultant adduct.

Covalent bond formation is possibly facilitated by specific DNA sequence contexts, which are more likely to be present in longer DNA fragments-potentially explaining the observed size-dependent effect. This observation does not rule out the possibility of covalent interaction, particularly if only one strand of the cross-linked DNA is covalently bound to PyDau, while the other strand is retained through strong non-covalent interactions. Moreover, covalent bond formation itself may be reversible over a slow timescale. Such a slow, reversible dissociation of the DNA–PyDau complex would still permit the anthracycline derivative to exert its cytotoxic effects by interfering with essential DNA-dependent processes such as replication and transcription.

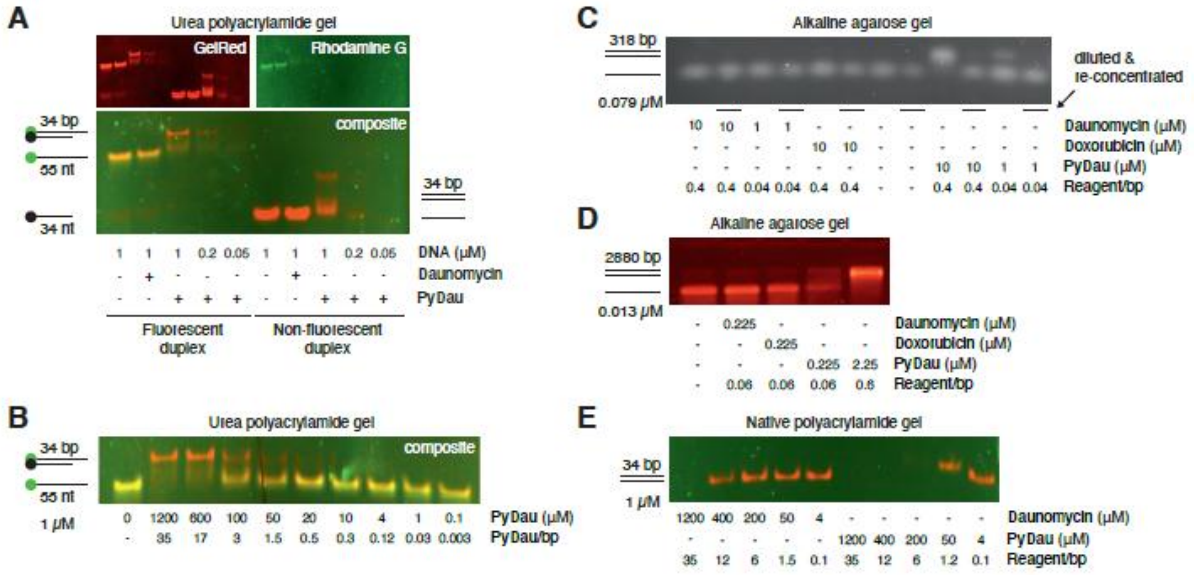

Supplementary Figure S4. PyDau cross-links the two strands of dsDNA in a denaturation resistant way.

A. Migration patterns of short (35 bp) internally quenched fluorescent dsDNA and its non-tagged 35 bp variant in urea denaturing gel. The duplex DNA constructs were incubated at different DNA concentrations in assay buffer either alone (control) or with DAU or PyDau at 37°C for 5 hours at constant reagent excess (14.7/bp of DNA), then applied to UREA denaturing gel electrophoresis, stained by GelRed, and scanned at two channels (Fluorescein epi-green and GelRed). Lower panel: Lane 1 and 6, the reagent-free (mock) controls at 1 µM DNA; lanes 2 and 7, Daunorubicin reaction at 1 µM; lanes 3-4-5 and 8-9-10 PyDau reactions at 1.0 µM, 0.2 µM, and 0.05 µM DNA concentrations. A new, urea resistant, low mobility band (corresponding to cross-linked DNA) was formed with PyDau, with both constructs. The highest mobility band (representing the quencher strand with the fluorescent construct) clearly disappears, and the produced lowest mobility material (a cross-linked dsDNA) remains non-fluorescent as the two strands could not separate in urea denaturing gel. DAU behaves like the control. The two upper panels show the Rhodamine Green and the GelRed signals separately.

B. Analysing the cross-linking potential of PyDau in denaturing condition at constant DNA (1 µM) and increasing (0.1-1200 µM) PyDau concentrations using the 35 bp internally quenched dsDNA construct.. The short fluorescent DNA construct was incubated in assay buffer (control) or with PyDau for 5 hours, then applied to UREA denaturing gel electrophoresis, stained by GelRed, and scanned for fluorescence (Fluorescein epi-green and GelRed) at different reagent concentration/excess and analysed. A non-fluorescent, low mobility band appears with increasing PyDau concentration/excess, indicating interstrand cross-linking and quenching of the intercalating dye (values are displayed at the bottom).

C. The cross-linking potential of PyDau with a medium-sized (318 bp) dsDNA at two different (10 and 1 µM) reagent concentrations. After 5 hours incubation at 37 °C in assay buffer the excess reagent was removed by phenol-chloroform purification and the DNA was applied to alkaline gel electrophoresis followed with GelRed staining and scanning. A control study where the purified DNA was dialysed prior to electrophoresis was also performed. The shifted band observed only with PyDau, but not with DAU and DOX. Black lines mark samples that were

extensively dialyzed before gel electrophoresis. An almost complete reduction of the low mobility band for PyDau is observed.

D. The cross-linking potential of PyDau with plasmid-sized (opened pUCHSO, 2880 bp) dsDNA. Incubation, phenol-chloroform purification, alkaline gel electrophoresis and detection was done as above (C). Full binding (a shifted band) is observed for PyDau at 2.25 μM (reagent excess of 0.06), while still significant amount of the DNA is cross-linked at 0.225 μM (reagent excess 0.006). No gel-shift is observable for DAU and DOX.

E. PyDau and DAU exhibit strong DNA binding under native conditions, but DNA cross-linking is observed exclusively with PyDau. A 34 bp fluorescently labeled dsDNA construct was incubated with increasing concentrations of DAU or PyDau, then analyzed by native PAGE. PyDau induced the appearance of a low-mobility band at significantly lower concentrations than in denaturing conditions (panel B), while DAU failed to produce such a shift. Complete loss of fluorescence occurred at ~6× excess PyDau, compared to 35× excess for DAU, indicating stronger and more efficient intercalation and cross-linking by PyDau.

Supplementary Table S3. Mortality rates and survival of free PyDau treated FVB mice.

	1 mg/kg	0.5 mg/kg	0.25 mg/kg	0.17 mg/kg	0.1 mg/kg	0.05 mg/kg
Mortality (%)	100	100	0	0	0	0
Survival (days)	4	11	>30	>30	>30	>30

Supplementary Table S4. Mortality rates and survival of LiPyDau treated FVB mice.

	1.5 mg/kg	0.5 mg/kg	0.4 mg/kg	0.35 mg/kg	0.3 mg/kg
Mortality (%)	0	0	0	0	0
Survival (days)	>10	>10	>10	>10	>10

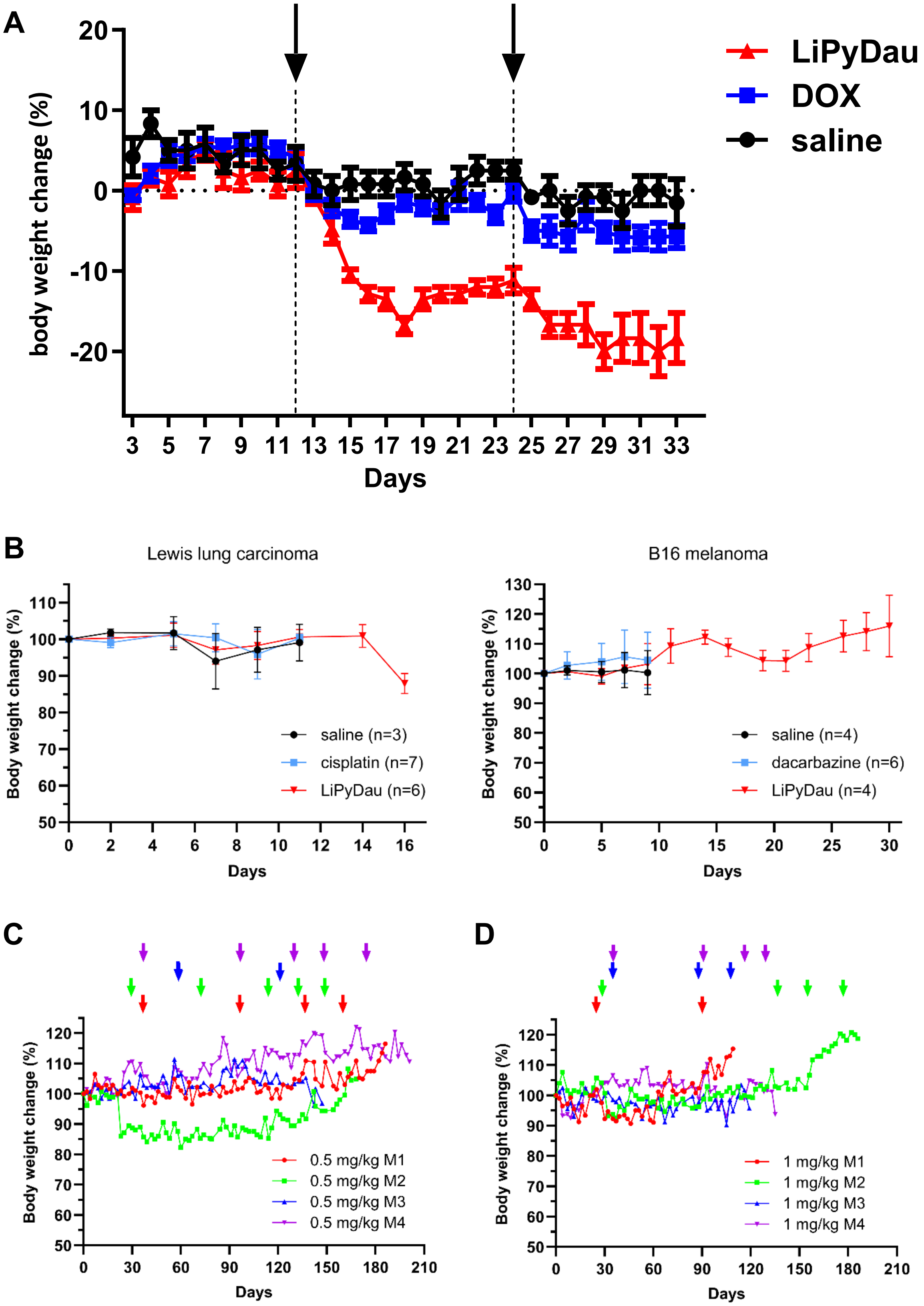

Supplementary Figure S5. Body weight changes related to treatment in different tumor models and mouse strains.

A. Changes in body weight of saline, DOX (5 mg/kg) and LiPyDau (1 mg/kg) treated BALB/c mice.

B. Changes in body weight of tumor-bearing C57BL/6 mice treated with saline, cisplatin (5 mg/kg), dacarbazine (70 mg/kg) or LiPyDau (1 mg/kg, single dose given on day 0).

C. Changes in body weight of FVB mice treated with three injections of LiPyDau at 0.5 mg/kg, administered at each tumor relapse (indicated by arrows). Body weight data corresponding to Figure 5; colors indicate the same mice shown in the corresponding panels of Figures 5 and S5).

D. Changes in body weight of FVB mice treated with two injections of 1 mg/kg LiPyDau (lower panel), administered at each tumor relapse (indicated by arrows). Body weight data corresponding to Figure 5; colors indicate the same mice shown in the corresponding panels of Figures 5 and S5).

Supplementary Figure S6. Chronic toxicity of LiPyDau treatment

Organoids derived from KB1P tumors[21] were engrafted into the 4th mammary fat pad of syngeneic FVB/N female mice. Mice developing mammary tumors (~100 ± 25 mm3) within 50 days following inoculation (3/8) were treated with LiPyDau. A single dose treatment with 1.25 mg/kg LiPyDau completely eradicated the organoid-derived tumors without any sign of toxicity (Figure 5G). Mice were sacrificed 600 days after tumor engraftment, and the liver, kidneys, spleen, heart, lungs, brain were subjected to histopathological analysis, along with an age-matched control. The myocardium did not show lesions in any animal, regardless of group assignment. In the lungs of all animals mild to moderate lymphoplasmacytic perivascular infiltration was evident and in the treated animals bronchioloalveolar adenomas were detectable. The control animal presented with multiple microgranulomas, few areas of hepatic necrosis, and mild peribiliary lymphocytic infiltration in the liver. The other animals showed few microgranulomas and mild peribiliary lymphocytic infiltration. Three out of four animals presented with scattered to mild amyloidosis of the renal glomeruli. All animals showed moderate amounts of hemosiderin and mild extramedullary hematopoiesis in the spleen. In the brain of all evaluated animals mild to moderate vacuolation of the white matter, predominantly in the striatum, thalamus, brainstem, and cerebellum was evident. All lesions detected were interpreted to be age associated.

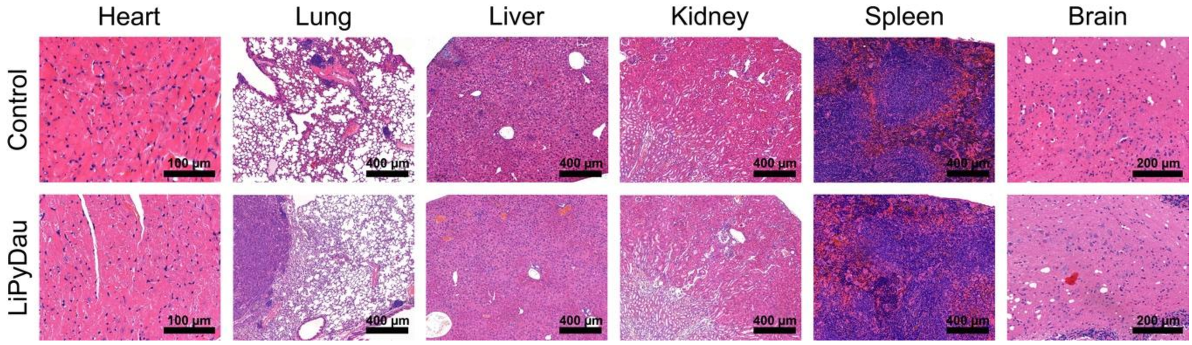

Figure S6. Hematoxylin and Eosin (H&E) staining of major organs. Representative sections of the heart, lung, liver, kidney, spleen and brain from 600-day-old untreated control mice (top row), and LiPyDau-treated mice (bottom row) are shown (see Figure 5.)

Supplementary Figure 7. Acute toxicity of LiPyDau treatment.

The pharmacokinetic study shown in Figure S10 provided samples to evaluate the short-term toxicity associated with a single curative dose of LiPyDau. Tumors were induced by orthotopic transplantation of Brca1$^{-/-}$;p53$^{-/-}$ FVB mouse mammary tumor fragments into the mammary fat pad of wild-type FVB mice. Once tumors reached approximately 200 mm³, mice were treated with LiPyDau. Animals were sacrificed at 0, 5 minutes, 1 hour, 1 day, 3 days, and 7 days post-treatment.

A detailed pathological analysis of major organs (heart, liver, kidney, lung, and spleen) at day 7 revealed no histological abnormalities or treatment-related damage, suggesting that LiPyDau is well tolerated at the therapeutic dose.

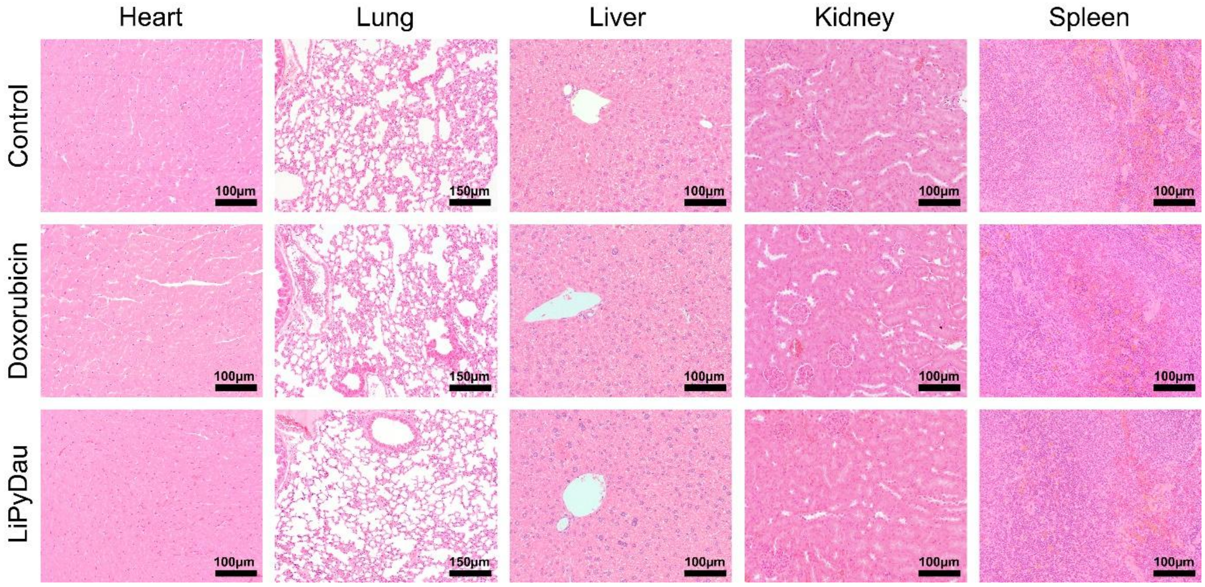

Figure S7A. Hematoxylin and Eosin (H&E) staining of major organs. Representative sections of the heart, lung, liver, kidney, and spleen from untreated control mice (top row), doxorubicin (DOX)-treated mice (middle row), and LiPyDau-treated mice (bottom row) are shown.

No lesions were observed in the myocardium or lungs of any animal, regardless of treatment group. In the control group, a few animals exhibited scattered microgranulomas and single-cell necrosis in the liver; similar findings were observed in the DOX- and LiPyDau-treated groups. One LiPyDau-treated animal displayed mild focal hepatic necrosis. Mild, focal perivascular lymphocytic infiltration in the kidney was observed in one animal each from the DOX and LiPyDau groups. In the spleen, all animals showed moderate hemosiderin accumulation and extramedullary hematopoiesis. The lesions were infrequent and limited to individual animals; therefore, they were considered incidental and unrelated to the treatment.

Given the well-known cardiotoxicity linked to anthracycline-based therapies, we also evaluated potential early cardiac damage by measuring serum cardiac troponin I levels using a high-sensitivity mouse assay [10](minimum detection limit: 0.156 ng/mL). All treated animals, including those receiving LiPyDau and free DOX, had troponin levels below the detection limit at all examined time points (0, 24 and 168 h). In contrast, cardiac troponin I levels were markedly increased in mice used as positive control, which were subjected to permanent ligation of the left coronary artery, confirming the assay's sensitivity and reliability.

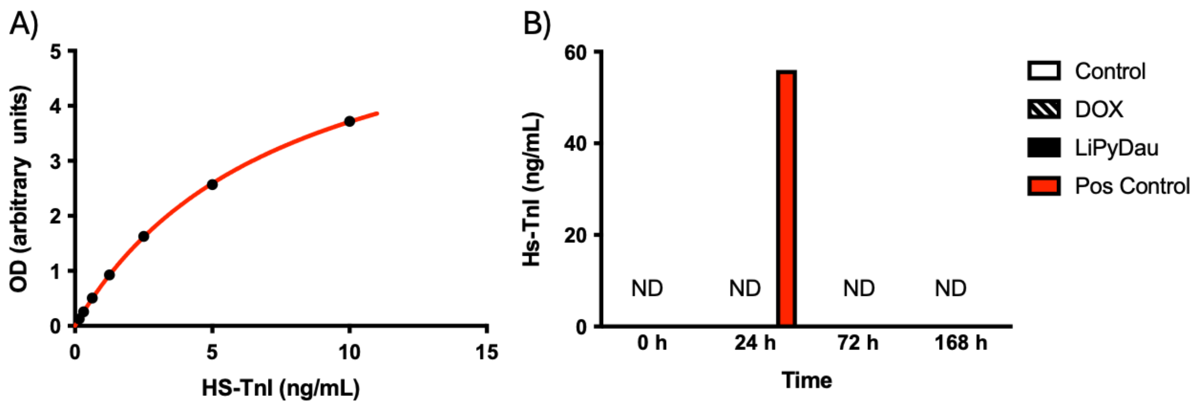

Figure S7B: Assessment of cardiotoxicity using high-sensitivity Troponin I ELISA reveals no detectable injury following LiPyDau treatment.

A. Calibration curve of the Hs-TnI ELISA assay showing linear detection in the range of 0.156–10 ng/mL.

B. Serum troponin I levels in untreated control mice and in mice treated with 5 mg/kg DOX or 1 mg/kg LiPyDau. No detectable (ND) troponin I was measured at any time point up to 7 days post-treatment. In contrast, mice subjected to left anterior descending coronary artery ligation (positive control) showed a robust increase in serum TnI levels 24 hours after the procedure, confirming the sensitivity and reliability of the assay.

Taken together, the histopathological analysis and high-sensitivity troponin I assay confirm that neither LiPyDau nor doxorubicin induced measurable acute cardiac injury following a single administration. Importantly, LiPyDau exhibited superior tumor-suppressive activity without detectable cardiotoxicity.

Supplementary Figure S8. *In vitro* drug release profile of LiPyDau under tumor-relevant conditions.

The *in vitro* drug release (IVR) studies were conducted in a simulated tumor microenvironment using ammonium chloride-containing buffer, following the methodology described by Silverman and Barenholz, who demonstrated enhanced release of doxorubicin from Doxil® under similar conditions[7]. IVR was investigated using HPLC-SEC to quantify the fraction of encapsulated PyDau over time. IVR was measured by incubating LiPyDau in a buffer containing 50 mM ammonium chloride, 10 mM histidine, and 10% sucrose at 37 °C. After 48 hours, 95% of the drug remained encapsulated, indicating limited release under these conditions. These results indicate that the release of drug from LiPyDau is not primarily driven by ammonium ion gradients, unlike in the case of liposomal doxorubicin.

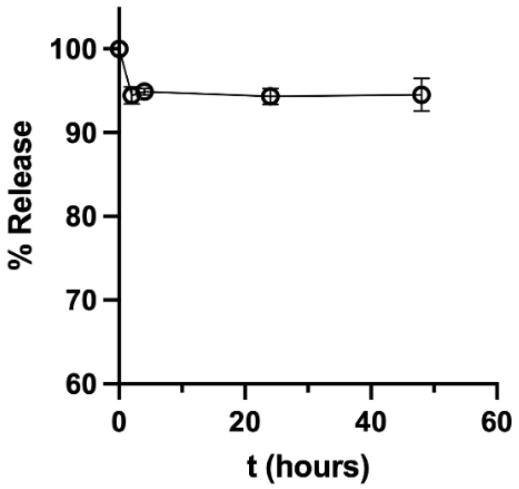

Supplementary Figure S8 ***In vitro* drug release** in ammonium chloride/histidine/sucrose buffer over 48 hours at 37 °C, showing the percentage of PyDau remaining encapsulated. The *in vitro* drug release from liposomes to the simulated tumor environment was assessed by separating encapsulated and free PyDau using high-performance liquid chromatography with size exclusion chromatography (HPLC-SEC). Measurements were performed on a Jasco HPLC system (Jasco Inc., Japan) equipped with a PU-2089 pump and a UV-2075 UV-Vis detector. Separation was achieved using Tricorn 5/100 glass columns (GE Healthcare, Sweden) packed with Sepharose CL-6B, with PBS as the eluent at a flow rate of 0.25 mL/min. PyDau was detected at 485 nm, its absorbance maximum, previously determined using a Hewlett-Packard 8453 diode array spectrophotometer (Hewlett-Packard, USA) with a 0.1 cm pathlength quartz cuvette maintained at 25 °C. For *in vitro* release studies, liposomes were diluted 1:5 in a buffer containing 50 mM ammonium chloride, 10 mM histidine, and 10% sucrose, and incubated at 37 °C for 48 hours.

Supplementary Figure S9. Plasma stability of LiPyDau

Plasma stability was assessed by incubating LiPyDau in 50% human plasma (diluted with 0.9% NaCl), and using a 9-fold dilution. At defined time points (0, 2, 4, 24, and 48 h), samples were analyzed by HPLC-SEC. The percentage of encapsulated PyDau was calculated as the ratio of encapsulated drug at each time point to the amount at time zero (T_0).

The results show a gradual decrease in encapsulated PyDau, reaching 72% of the initial concentration after 48 hours. The plasma stability of LiPyDau is slightly lower than that of liposomal doxorubicin, as reported by Shibata et al.[8], likely due to differences in the chemical properties and encapsulation state of the drug. However, the *in vivo* circulation half-life of LiPyDau is shorter than its *in vitro* plasma half-life, indicating that plasma stability is not the rate-limiting factor in its clearance. This is further supported by the *in vivo* tumor concentration profile, which peaks at around 33 hours.

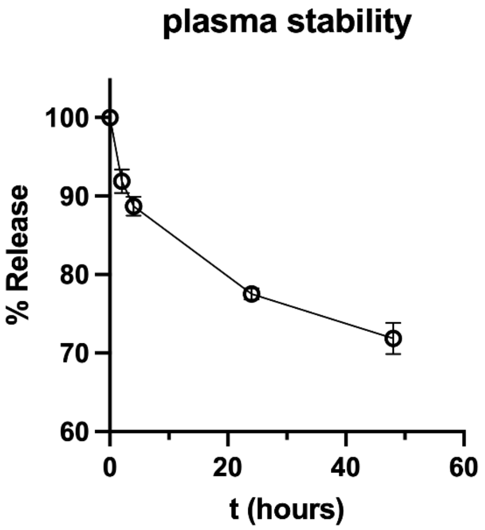

Figure S9. Plasma stability of LiPyDau in 50% human plasma over 48 hours, showing a gradual decrease in the encapsulated PyDau fraction. Encapsulated PyDau content was quantified using HPLC-SEC.

Supplementary Figure S10. Pharmacokinetic profile of LiPyDau in plasma and tumor tissue.

To assess intratumoral distribution, we designed a pharmacokinetic study. Briefly, tumors were induced by orthotopic transplantation of Brca1$^{-/-}$;p53$^{-/-}$ FVB mouse mammary tumor fragments into the mammary fat pad of wild-type FVB mice. Once tumors reached approximately 200 mm³, mice were treated with LiPyDau. Animals were sacrificed at 0, 5 minutes, 1 hour, 1 day, 3 days, and 7 days post-treatment. Serum and intratumoral levels of PyDau were quantified by mass spectrometry (MS).

As shown in Figure S10A, following intravenous injection of the curative dose of LiPyDau, PyDau plasma levels decayed slowly, reaching an AUC value of 1.05*10^{7} ng*h/ml. As expected based on the EPR effect, elevated plasma PyDau levels resulted in a significant tumoral drug load (Figure S10B).

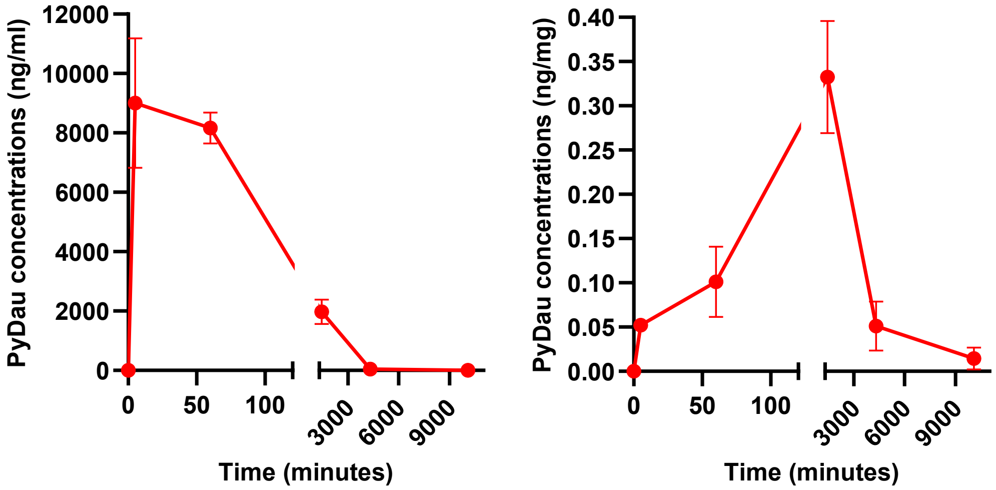

Figure S10. Serum (A) and tumoral (B) PyDau concentrations in mice receiving a single dose of 1 mg/kg iv. LiPyDau treatment. PyDau levels were determined at the indicated time points by mass spectroscopy. Points represent means of triplicate aliquots from 3 mice per time point; error bars represent ± SD.

These results may be compared to those obtained with the PEGylated liposomal formulation of doxorubicin (PLD/Doxil®/Caelyx®) in the same mouse model, as reported in Furedi et al.[9]. As shown in Fig. 5 of Furedi et al[9], PLD achieved a significantly higher AUC than free doxorubicin, which was accompanied by a marked increase in intratumoral drug accumulation. Following PLD treatment, doxorubicin levels in plasma fell below detection between days 3 and 5, and were already undetectable in tumor tissue by day 5. In contrast, after LiPyDau administration, plasma PyDau levels dropped below the detection limit in between days 1 and 3, yet remained detectable in tumor tissue even 7 days post-treatment. PLD was administered at a dose of 8 mg/kg, whereas LiPyDau was given at only 1 mg/kg. Despite an eight-fold higher

injected dose of PLD (8 mg/kg vs. 1 mg/kg), LiPyDau achieved nearly 30% of PLD's peak serum concentration (9004 vs. 31,600 ng/mL) and approximately 23% of its AUC (1.05×10^7 vs. 4.47×10^7 ng·h/mL). Likewise, despite the eightfold difference in administered dose, the peak intratumoral concentration of PyDau reached 0.30–0.35 ng/mg, compared to approximately 1.4 ng/mg for DOX following PLD treatment.

Monitoring of plasma and intratumoral pharmacokinetics of PyDau

Brca1;p53-KO mammary tumor bearing mice were treated with 1 mg/kg LiPyDau and sacrificed at 0 (before treatment), 5 minutes, 1 hour, 1 day, 3 days, and 7 days post-treatment by cervical dislocation. Whole blood was collected by cardiac puncture, centrifuged at 2000 × g and 4°C for 10 minutes and the plasma supernatant was immediately collected. Acetonitrile (ACN) was added to the samples in 1:5 ratio (1 sample + 5 ACN) to precipitate the liposomes and free the PyDau content.

To measure intratumoral PyDau concentrations the tumors were removed and weighted shortly after euthanasia, minced in PBS, mixed thoroughly, centrifuged at 400 × g and room temperature, samples were taken from the supernatant and treated with ACN in 1:5 ratio. From both plasma and tumor samples 50 µl was used for measurement.

Mass spectrometric measurements were performed on a Sciex 6500QTrap (Sciex, Framingham, MA, USA) hybrid quadrupole-ion trap mass spectrometer equipped with Turbo-V ion source. HPLC separations were carried out on an Agilent 1100 system consisting of a binary pump, an autosampler and a column compartment. Analyst 1.6.3. (Sciex, Framingham, MA, USA) software was used for controlling the instrument and for data processing. Quantitation was performed by using the Sciex Multiquant software.

HPLC separation of the samples was performed on a Kinetex EVO C18 (50 x 2.1 mm, 5µm). The mobile phases were: water containing 0.1% formic acid (eluent A) and acetonitrile containing 0.1% formic acid (eluent B). The separation was performed in gradient elution mode. The initial eluent B composition was 5%. A linear gradient was applied to reach the 95% of eluent B by 5 min and kept there for 1.5 min. The initial solvent composition was set at 0.5 min and the equilibration time was 3 min. The overall run time was 10 min. The flow rate was 0.6 mL/min and the injection volume was 5 µL. The column temperature was ambient.

MS detection in positive ion MRM mode was applied for quantitation of the target molecule. Source parameters were: curtain gas (CUR), nebulizer gas (GS1) and drying gas (GS2) values were set at 40, 40 and 40 arbitrary unit, respectively. The spray voltage was 5500V. Source temperature was set at 450°C. Declustering potential was set to 100V. The PyDau was

detected in MRM mode with transitions of 580.2/321 (quantifier) and 580.2/363 (qualifier) (Q1/Q3) with dwell time of 100 ms. Collision energy was 30eV.

References

1. Shoemaker RH. The NCI60 human tumour cell line anticancer drug screen. Nat Rev Cancer. 2006;6:813–23.

2. Varbanov HP, Kuttler F, Banfi D, Turcatti G, Dyson PJ. Repositioning approved drugs for the treatment of problematic cancers using a screening approach. PLoS One. 2017;12:e0171052.

3. Póti Á, Gyergyák H, Németh E, Rusz O, Tóth S, Kovácsházi C, et al. Correlation of homologous recombination deficiency induced mutational signatures with sensitivity to PARP inhibitors and cytotoxic agents. Genome Biol. 2019;20:240.

4. Fusani L, Palmer DS, Somers DO, Wall ID. Exploring Ligand Stability in Protein Crystal Structures Using Binding Pose Metadynamics. J Chem Inf Model. 2020;60:1528–39.

5. Wang AH, Gao YG, Liaw YC, Li YK. Formaldehyde cross-links daunorubicin and DNA efficiently: HPLC and X-ray diffraction studies. Biochemistry. 1991;30:3812–5.

6. Post GC, Barthel BL, Burkhart DJ, Hagadorn JR, Koch TH. Doxazolidine, a Proposed Active Metabolite of Doxorubicin That Cross-links DNA. J Med Chem. 2005;48:7648–57.

7. Silverman L, Barenholz Y. In vitro experiments showing enhanced release of doxorubicin from Doxil® in the presence of ammonia may explain drug release at tumor site. Nanomedicine: Nanotechnology, Biology and Medicine. 2015;11:1841–50.

8. Shibata H, Izutsu K, Yomota C, Okuda H, Goda Y. Investigation of factors affecting*in vitro*doxorubicin release from PEGylated liposomal doxorubicin for the development of*in vitro*release testing conditions. Drug Development and Industrial Pharmacy. 2015;41:1376–86.

9. Füredi A, Szebényi K, Tóth S, Cserepes M, Hámori L, Nagy V, et al. Pegylated liposomal formulation of doxorubicin overcomes drug resistance in a genetically engineered mouse model of breast cancer. Journal of Controlled Release. 2017;261:287–96.

10. Nicol M, Sadoune M, Polidano E, Launay JM, Samuel JL, Azibani F, et al. Doxorubicin-induced and trastuzumab-induced cardiotoxicity in mice is not prevented by metoprolol. ESC Heart Failure. 2021;8:928–37.
